# Intergenerational inheritance of high fat diet-induced cardiac lipotoxicity in *Drosophila*

**DOI:** 10.1038/s41467-018-08128-3

**Published:** 2019-01-14

**Authors:** Maria Clara Guida, Ryan Tyge Birse, Alessandra Dall’Agnese, Paula Coutinho Toto, Soda Balla Diop, Antonello Mai, Peter D. Adams, Pier Lorenzo Puri, Rolf Bodmer

**Affiliations:** 1 Development, Aging and Regeneration Program, Sanford-Burnham-Prebys Medical Discovery Institute, 10901 N. Torrey Pines Road, La Jolla, CA 92037 USA; 2 Sapienza Università di Roma, 00185, Rome, Italy; 3 IRCCS Fondazione Santa Lucia, 00142, Rome, Italy; 4 Present Address: Biocompatibles Inc., 300 Four Falls Corporate Center, 300 Conshohocken State Road, West Conshohocken, PA 19428-2998 USA; 5 Present Address: Whitehead Institute for Biomedical Research, 455 Main Street, Cambridge, MA 02142 USA

## Abstract

Obesity is strongly correlated with lipotoxic cardiomyopathy, heart failure and thus mortality. The incidence of obesity has reached alarming proportions worldwide, and increasing evidence suggests that the parents’ nutritional status may predispose their offspring to lipotoxic cardiomyopathy. However, to date, mechanisms underlying intergenerational heart disease risks have yet to be elucidated. Here we report that cardiac dysfunction induced by high-fat-diet (HFD) persists for two subsequent generations in *Drosophila* and is associated with reduced expression of two key metabolic regulators, adipose triglyceride lipase (ATGL/*bmm*) and transcriptional cofactor PGC-1. We provide evidence that targeted expression of ATGL/*bmm* in the offspring of HFD-fed parents protects them, and the subsequent generation, from cardio-lipotoxicity. Furthermore, we find that intergenerational inheritance of lipotoxic cardiomyopathy correlates with elevated systemic H3K27 trimethylation. Lowering H3K27 trimethylation genetically or pharmacologically in the offspring of HFD-fed parents prevents cardiac pathology. This suggests that metabolic homeostasis is epigenetically regulated across generations.

## Abstract (web summary)

Animal studies have shown that the nutritional status of parents can predispose the offspring to obesity and obesity-related diseases. Here the authors show that cardiac dysfunction induced by a high-fat diet persists for two generations in *Drosophila*, and that targeted expression of ATGL/*bmm* in the offspring, as well as inhibition of H3K27 trimethylation, is cardioprotective.

## Introduction

Heart failure is the main cause of mortality worldwide and is now further aggravated by the growing obesity epidemic. Considerable efforts have been made to identify the genetic basis of obesity. However, genome-wide association studies have rarely established causality between genetic variants and obesity or type 2 diabetes, despite the clearly established importance of heritability in the development of these ailments^1–3^. Thus, other forms of variation are likely involved, such as epigenetic factors, that may be able to confer changes to the genomic material to be inherited without altering the genetic code. In fact, compelling evidence indicates that the deleterious effects of maternal and paternal obesity are strongly linked to an increased risk for developing obesity-related pathologies, including, type 2 diabetes and cardiovascular disease in the offspring^4–9^. Based on epidemiological studies, Barker and others^10^ formulated the hypothesis that nutritional quality during gestation may result in what is known as fetal programming, thereby predisposing the offspring to metabolic and cardiovascular disorders later in life. Several animal model studies, conducted in fruit flies, mice, and non-human primates, provided compelling evidence for this hypothesis. These studies collectively show that progeny of parents exposed to a high-fat diet (HFD) and/or high-sugar diet (HSD) during gestation are more susceptible to developing obesity and obesity-related diseases as adults^11^. However, the mechanistic basis for this phenomenon remains elusive.

To investigate whether parental exposure to a HFD increases the risk of obesity and cardiac lipotoxicity in the progeny, we used the fruit fly *Drosophila* as a model system. *Drosophila* with its short generation time is well suited for transgenerational studies and is well established as a powerful tool for discovering conserved gene networks in cardiac development, function, and metabolism^12–14^. In *Drosophila*, both high-fat and high-sugar diets increase fat storage and perturb metabolic homeostasis that are classic hallmarks of metabolic syndrome and diabetic cardiomyopathy^15–17^. Additionally, recent studies suggest that these extreme diets may have long-lasting effects over multiple generations^18,19^.

Here, we establish a *Drosophila* model to study the intergenerational inheritance of HFD-induced lipotoxic cardiomyopathy and present first evidence of a mechanism underlying this phenomenon. Our data indicate that reduced expression levels of *adipose triglyceride lipase* (*ATGL*; *brummer (bmm)* in *Drosophila*) and *peroxisome proliferator-activated receptor-γ coactivator 1α* (*PGC-1α, PGC-1* in *Drosophila*) are closely associated with intergenerational lipotoxic cardiomyopathy. Furthermore, we find that cardio-protection can be achieved by cardiac or systemic *bmm* overexpression, which can lead to re- and even pre-programming of the metabolic state of a developing or adult individual. Finally, we demonstrate that intergenerational inheritance of HFD-induced lipotoxic cardiomyopathy coincides with elevation of systemic levels of trimethylated H3K27 (H3K27me3). Importantly, overexpression of the H3K27me3 demethylase, UTX, or pharmacological inhibition of the H3K27 methyltransferase, polycomb repressive complex 2 (PRC2) enzymatic subunit EzH2, could reverse the elevated H3K27me3 levels and prevent intergenerational transmission of parental HFD-induced cardiac lipotoxicity.

## Results

### HFD-induced cardiac lipotoxicity is inherited

**Fig. 1:**
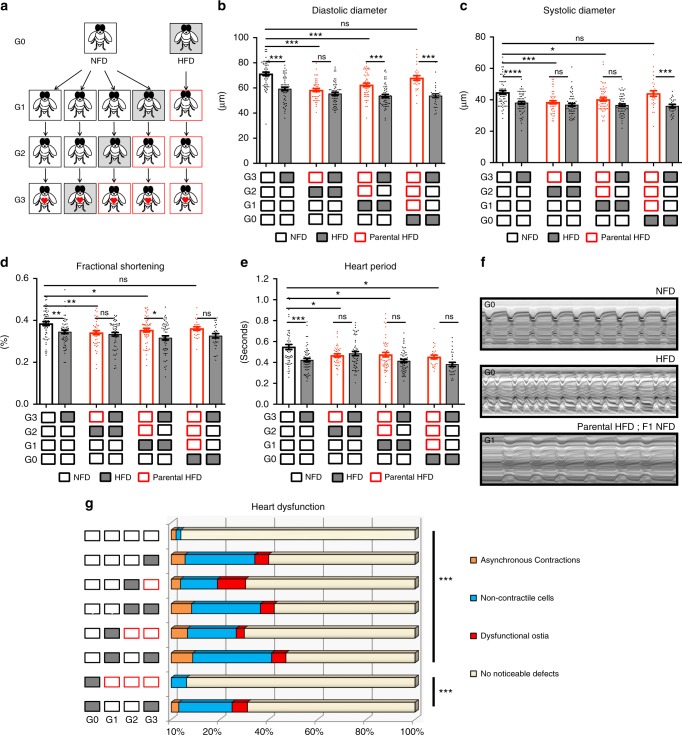
Parental HFD exposure causes intergenerational heart dysfunction. **a** Diagram with generation of control flies (*w*^*1118*^) fed a normal food diet (NFD; open boxes) or a high-fat diet (HFD; gray boxes). Boxes outlined in red highlight the generations on NFD after the original exposure to a HFD, and correspond to the bars with red outline in all bar charts (parental HFD). Flies with red hearts indicate the experimental flies tested for changes in heart function **b**–**g** TAG levels (Fig. 2c) and *bmm* transcript levels (Fig. 2g). Note that only mated female adults were analyzed in this study. Diastolic diameters (**b**) systolic diameters (**c**), fractional shortening (**d**) and heart period (**e**) were measured in G3 adult flies (*n* = 25–55 flies pooled from at least 3 independent experiments). Statistical analysis were conducted by one-way ANOVA followed by Dunnett’s multiple comparisons post-test *p < 0.05, **p < 0.01,***p < 0.001, ns not significant. Graphs show mean ± SEM (error bars), overlaid with single data points. **f** Representative M-mode traces prepared from high-speed movies of semi-intact heart preparations on flies exposed to NFD, HFD, or parental HFD. **g** Acute and parental HFD causes a significant increase in cumulative heart dysfunctions. Heart phenotypes including conduction blocks, non-contractile regions, and dysfunctional inflow tracks were quantified (*n* *=* 24–30, ***p < 0.001, chi-square test). Of note, in contrast to all other heart parameters examined, heart period does not return to normal in the 3rd generation of offspring, suggesting that some aspects of cardiac pathology may last longer than two progeny generations after parental HFD

To investigate the consequences of a parental HFD, we evaluated cardiac and metabolic function in three generations of female offspring fed a normal food diet (NFD, diagram in Fig. 1a). Strikingly, we found that the effects of a HFD on heart function were evident not only in the HFD-fed parents themselves^15^, but also in their first- and second-generation progeny raised on NFD. Both progeny generations displayed dramatic detrimental changes in heart function, including decreased diastolic and systolic diameters, reduced fractional shortening, reduced heart beat length (heart period), and increased incidence of non-contractile myocardial cells, partial conduction blocks, and dysfunctional inflow valves, called ostia (Fig. 1b–g). The impaired heart phenotypes returned to normal in the third generation of offspring on a NFD (Fig. 1b–d, g).

**Fig. 2:**
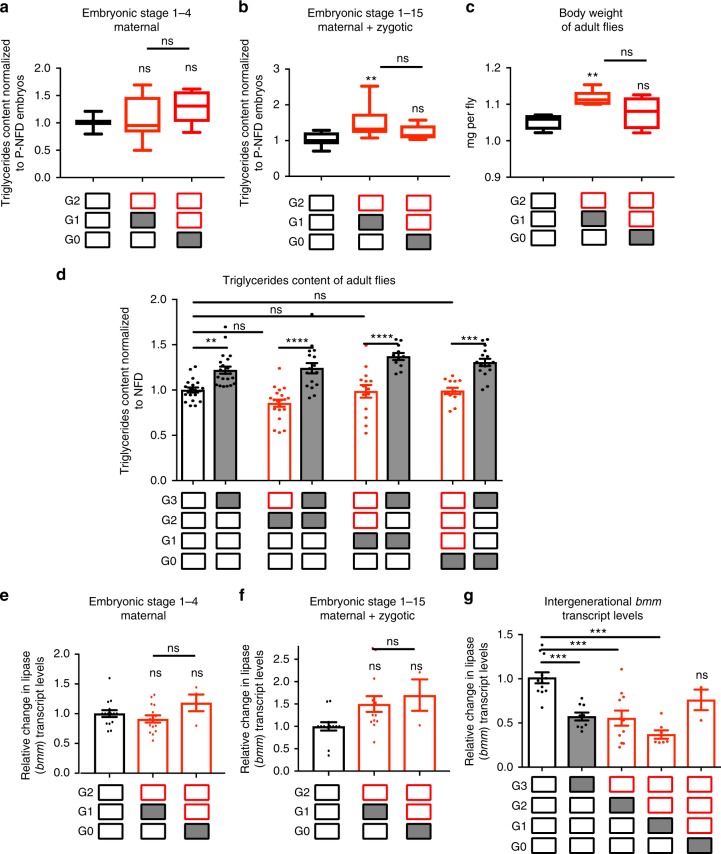
Parental HFD causes intergenerational elevation of lipid content and decreased *bmm* RNA levels. TAG content of **a** early-stage embryos (stages 1–4, maternal contribution only, before zygotic transcription starts), and **b** early- and late-stage embryos (stages 1–15, maternal and zygotic contribution) from parents and grandparents fed NFD or HFD (*n* = 5–15 samples from 4 independent experiments). **c** Body weight of adult female flies after acute or parental exposure to NFD or HFD, *n* = 4–6 samples per condition, consisting of 20 pooled flies each. Box plots show the median (center horizontal line), interquartile range (box), and 5th to 95th percentiles (whiskers). **d** TAG content of three generations of adult progeny after acute or (grand)parental exposure to NFD or HFD, measured in G3; *n* = 12–22 samples consisting of a pool of 3 adult female flies for each condition. TAG values are relative to body weight and normalized to control NFD. **e** Maternally contributed and **f** post-zygotically transcribed *bmm* transcript levels in embryos from flies fed NFD, HFD, or parental HFD (*n* = 3–13 samples from 3 independent experiments). **g** Normalized ATGL/*bmm* RNA levels in G3 adult flies after acute or (grand)parental exposure to HFD; *n* = 3–12, each sample was prepared by homogenizing 10 flies. TAG content (**d**) and *bmm* transcript levels (**g**) were measured in whole female-mated flies and normalized to control NFD-fed flies. Samples were compared to NFD, otherwise depicted; **p < 0.01, ***p < 0.001, ****p < 0.0001, ns not significant using one-way ANOVA followed by Sidak’s multiple comparisons test. Graphs in (**d**–**g**) show mean ± SEM (error bars), overlaid with single data points

HFD also caused increased fat accumulation in the parents, as observed previously^15,16^, which persisted in the progeny of HFD-fed parents, manifested as higher fat content in embryos at stages 1–15 (early to late stage embryogenesis) and subsequently as bigger and heavier adult offspring (Fig. 2b–c). The increase in body mass is likely because HFD augments growth-promoting Target of rapamycin signaling^15^. However, first progeny generation adults did not exhibit an increase in fat content relative to body weight (Fig. 2d). In addition, second-generation progeny of HFD-fed parents raised on a NFD presented no significant increase in fat content at embryo stages 1–15 or as adults, and were no longer significantly heavier (Fig. 2b–d). Interestingly, the offspring’s increase in fat accumulation at late embryonic stages was not due to increased maternal contribution, since early embryos (stages 1–4, before the start of zygotic transcription) exhibited no difference in fat content when parents or grandparents were fed a HFD, compared to embryos from NFD-fed parents (Fig. 2a).

### HFD induces intergenerational reduction of ATGL expression

Previous work suggested that HFD reduces the expression of *bmm* in *Drosophila*^15^. Therefore, we asked whether parental exposure to a HFD affected *bmm* transcript levels in subsequent generations. We found no differences in the levels of maternally deposited *bmm* transcripts when early, pre-zygotic embryos from HFD-fed parents or grandparents were analyzed, in comparison to embryos from NFD-fed flies across generations (Fig. 2e). In addition, *bmm* transcript levels of embryos from HFD-fed parents or grandparents were also unchanged later in development (Fig. 2f). In contrast, we found that parental HFD decreased total adult *bmm* transcript levels for two generations, returning to normal only in the third generation (Fig. 2g).

The persistent effects of a HFD in one generation on future generations can be explained in two ways: (1) an ‘intergenerational’ effect that lasts for two generations following exposure of the parents (G0) to the initial dietary insult, presumably due to the direct exposure (within the parent) of the first-generation (G1) oocyte/egg and its germplasm (future G2); and (2) a ‘transgenerational’ effect that persists up to or beyond the third generation^11^. Our data suggest that parental HFD induces an ‘intergenerational’ effect (Figs. 1g and 2g), since both heart dysfunction and reduction of *bmm* transcript levels persist for only two generations after parental exposure to a HFD.

### Cardiac lipotoxicity caused by *PGC-1* depletion is inherited

In order to validate the intergenerational model of HFD-induced lipotoxic cardiomyopathy, we genetically induced lipid accumulation and tested the effects on heart function and metabolism in the next generation. For this purpose we used the previously characterized heterozygous *PGC-1/spargel* mutant flies (*PGC-1*^*XP*^)^16^. PGC-1 is a potent transcriptional co-activator that mediates mitochondrial activity in response to insulin signaling^20^. Reduction of *PGC-1* function mimics the effects of a HFD on heart function and lipid content by acting downstream of *bmm* in *Drosophila*^16^. Specifically, heart dysfunction phenotypes of *PGC-1*^*XP*^ heterozygotes (and of another PGC-1 allele) have previously been found to be the same as those of heart-specific *PGC-1* knockdown^16^. Additionally, heart dysfunction of *PGC-1*^*XP*^ heterozygotes was rescued by a genomic construct (*PGC-1*^*GR*^^20^), as reported by Diop et al.^16^, thus providing strong evidence that cardiomyopathy observed in these *PGC-1*^*XP*^ heterozygotes is due to loss of *PGC-1* function.

**Fig. 3:**
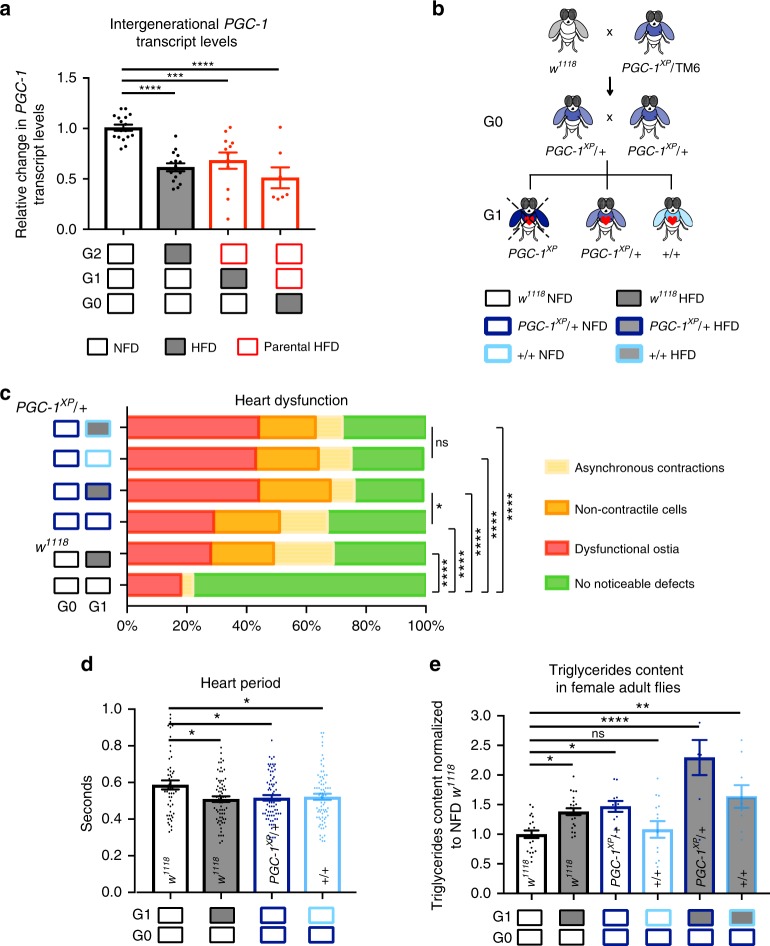
Depletion of *PGC-1* causes changes in fat content and heart function in the next generation. **a** *PGC-1* transcript levels of whole flies fed NFD, acute HFD, or parental HFD. *PCG-1* mRNA levels are normalized to *rp49* (*n* = 7–17 samples per condition consisting of more than 6 female flies each from 4 independent experiments). **b** Schematic representation of the genetic crosses performed to study the influence of partial depletion of *PGC-1* on heart function and TAG content in the next generation of female flies. *PGC-1* heterozygous mutants were crossed to control flies (*w*^*1118*^) and the offspring (*PGC-1/+*) were collected and crossed again in order to generate G1 progeny consisting of *PGC-1*^*XP*^ homozygous mutants (larval/pupal lethal), *PGC-1*^*XP/+*^ heterozygous, and *wildtype* (+/+ for *PGC-1* locus). **c** Heart dysfunction phenotypes, i.e., partial conduction block, non-contractile regions, and dysfunctional ostia were quantified (*n* = 55 flies per genotype, ***p < 0.001, chi-square test). **d** Heart period was measured in G1 flies (*n* = 51–74 flies). **e** Whole flies TAG content normalized to *w*^*1118*^ NFD levels (*n* = 9–23 samples per condition consisting of 3 adult flies each). Note that while +/+ flies do not show a significant increase in TAG content, their response to HFD is exacerbated compared to *w*^*1118*^ flies on HFD. Statistical analysis in (**a**, **d**, **e**) were conducted by one-way ANOVA, followed by Dunnett’s multiple comparisons test *p < 0.05, **p < 0.01, ***p < 0.001, ****p < 0.0001 and ns not significant. Graphs in (**a**, **d**, **e**) show mean ± SEM (error bars), overlaid with single data points

Indeed, we found in control flies that HFD reduced transcript levels of *PGC-1* for two generations, suggesting that reduced *PGC-1* expression is also associated with intergenerational inheritance of HFD-induced lipotoxic cardiomyopathy (Fig. 3a), and may be sufficient for its persistent effect across generations. Thus, we proceeded to study the effect of parental *PGC-1* depletion on heart function of the offspring. *PGC-1*^*XP*^ heterozygous mutants (*PGC-1*^*XP*^*/+*) were outcrossed to control flies and then intercrossed (G0). Their progeny (G1) was assessed for heart function and lipid content (Fig. 3b). We found that parental *PGC-1* heterozygosity had a similar detrimental effect on heart function in the genetically wild-type progeny (+/+ for *PGC-1*), as on acute HFD or their *PGC-1*^*XP*^*/+* siblings; they all exhibited increased incidence of dysfunctional ostia, non-contractile myocardial cells, partial conduction block resulting in asynchronous contractions (Fig. 3c), and decreased heart period (Fig. 3d).

As reported previously, we found that *PGC-1*^XP^/+ flies have increased fat content, similar to HFD-fed flies^16^, but +/+ flies did not exhibit significant differences in triglyceride levels compared to NFD-fed flies. However, HFD triggered a stronger increase in fat content in these +/+ flies from *PGC-1*^*XP*^*/+* parents (6th bar, Fig. 3e) compared to control flies (2nd bar, Fig. 3e). These results provide genetic evidence to support the intergenerational inheritance of lipotoxic cardiomyopathy induced by a parental obesogenic metabolic state.

### *bmm* expression reverts inherited cardiac lipotoxicity

**Fig. 4:**
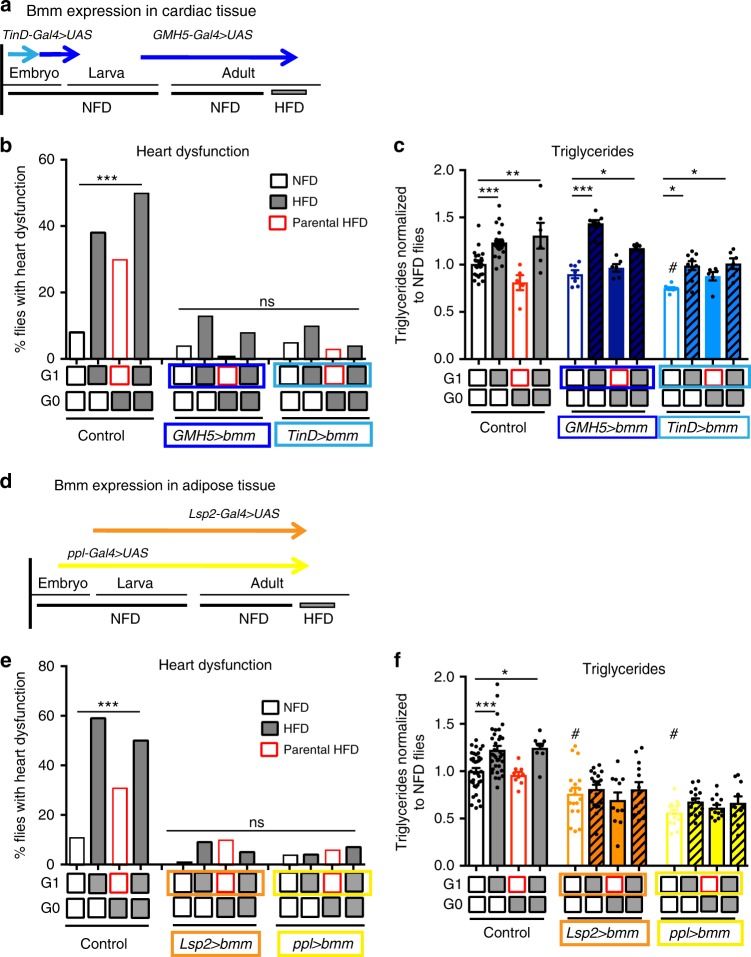
Tissue-restricted expression of *bmm* lipase protects the heart from parental or acute HFD. **a** Schematic illustration of the temporal expression of myocardial Gal4 drivers in the embryonic, larval, and adult stages. *TinD*-Gal4-driven expression (light blue arrow) is restricted to the dorsal mesoderm/cardiac progenitor cells at early to mid-embryogenesis^25^. The *GMH5*-Gal4 driver (dark blue arrow) is active during mid to late embryogenesis, and again throughout adulthood^48^. Gray box indicates 5 days of HFD exposure. **b** Cumulative incidence of heart defects and **c** TAG content in G1 upon parental or acute HFD (*n* = 24–30 flies and *n* = 6–23 samples respectively). **d** Schematic illustration of the temporal expression of adipose-specific Gal4 drivers. *ppl*-Gal4^27^ (yellow arrow) expression is restricted to the embryonic, larval, and adult stages, and *Lsp2*-Gal4 (orange arrow) is active during the larval and young adult stages^21^. **e** Cumulative incidence of heart defects and **f** TAG content in G1 (*n* = 24–30 flies and *n* = 9–36 samples respectively). Chi-square test was conducted to statistically analyze heart dysfunction. Experiments were repeated at least twice, and a representative experiment is shown. One-way ANOVA followed by Dunnett’s multiple comparisons test was conducted to analyze TAG content per genotype, *p < 0.05, **p < 0.01, ***p < 0.001 and ns not significant. Note that *TinD*>bmm, *Lsp2*>bmm and *ppl*>bmm presented reduce fat content compared to control flies fed NFD (^#^*p* < 0.05, two-tailed unpaired *t*-test). *w*^1118^ were used as controls. TAG content is expressed in mean ± SEM (error bars) and single data points were overlaid. See Supplementary Figure 1 for schematic diagram of the crosses and genetic controls. See Supplementary Figure 2 for driver specificity and Supplementary Figure 3 for additional heart parameters

Cardiac lipotoxicity has been found to be prevented by lipolysis induction upon genetically elevating *bmm* expression in *Drosophila*^15^, and manipulating ATGL in mammals and humans^21,22^. Since the intergenerational HFD-induced heart phenotypes are accompanied by reduced *bmm* expression, we used the GAL4/UAS system^23^ to overexpress *bmm* at different stages of development. We found that transgenic expression of *bmm* in the adult and embryonic myocardium, using the cardiac *GMH5-*Gal4 driver^24^, or the embryonic cardiac mesoderm *TinD*-Gal4 driver^25^, protected flies from HFD-associated cardiac dysfunction (Fig. 4a, b and Supplementary Figure 3a-e; also see Supplementary Figure 1 and Supplementary Figure 2 for genetic controls and driver specificity, respectively). Remarkably, expression of *bmm* in myocardial progenitors and adult cardiomyocytes protected the adult heart not only from the adverse effects of the parental HFD, but also from acute exposure to a HFD (Fig. 4b). In contrast, transgenic expression of *bmm* in adult and/or embryonic cardiomyocytes did not prevent the systemic increase in triacylglyceride (TAG) levels upon acute exposure to HFD (Fig. 4c). This suggests that autonomous expression of *bmm* in cardiomyocytes counteracts the effects of HFD and parental HFD in the heart. Moreover, it also implies that cardiac progenitors can be autonomously re- and even pre-programmed in the early embryo to protect against pathological heart phenotypes later in life.

### Adipose *bmm* expression reverts inherited cardiac lipotoxicity

In *Drosophila*, the major site for lipid storage is the fat body, which may systemically modulate cardiac lipotoxicity. We first validated the specificity of commonly used fat body drivers. By performing RNAscope® against *Gal4* and immunohistochemistry we found that both *Lsp2-Gal4*^26^ and *ppl-Gal4*^27^ drive strong expression in fat body of young flies; and while *Lsp2-Gal4* is specific for fat body, *ppl-Gal4* showed a mild expression in pericardial cells (Supplementary Figure 2c,d,k and m). Indeed, expression of *bmm* in the larval and adult fat body, using these two different drivers (*Lsp2*-Gal4 and *ppl*-Gal4), prevents heart dysfunction following parental or acute exposure to a HFD (Fig. 4d, e; and Supplementary Figure 3f-j. See also Supplementary Figure 1a-c for genetic controls). In line with these results, specific overexpression of *bmm* using *Lsp2-Gal4* or *ppl-Gal4* resulted in a moderate decrease of fat content and prevented HFD-induced fat accumulation (Fig. 4f). These results corroborate the association of metabolic imbalance and obesity to increased risk for heart dysfunction.

### Early embryonic *bmm* expression is cardioprotective

**Fig. 5:**
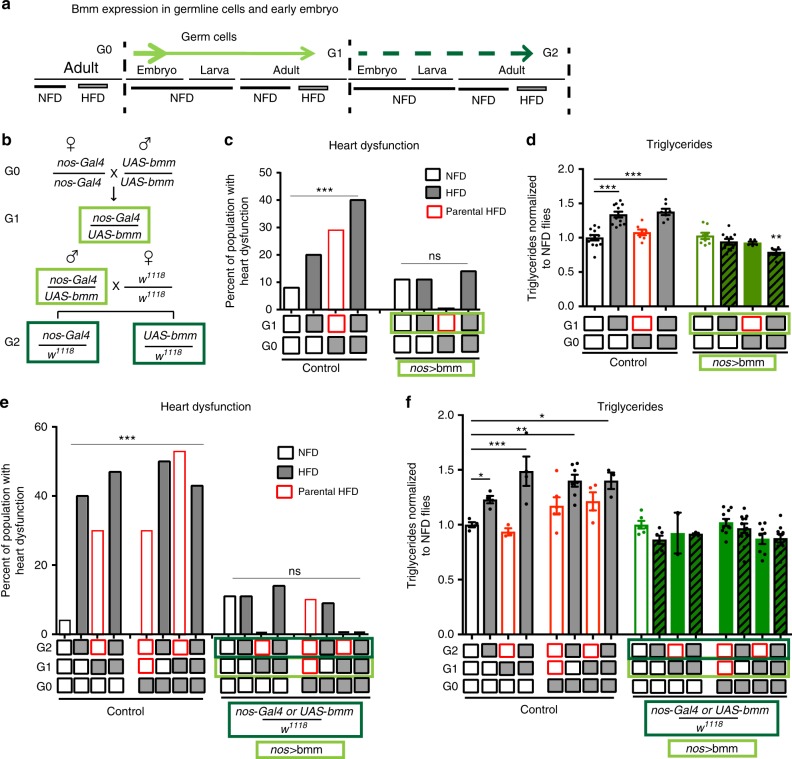
Early embryonic and future germ cell expression of *bmm* lipase protects the progeny and their offspring from the effects of parental or acute exposure to a HFD. **a** Schematic of the germline expression Gal4 driver, *nos*-Gal4 (green arrow). *nos*-Gal4 is expressed in the maternal germline and localized in the posterior region of the maturing oocytes, persists until the early embryonic stages to turn on UAS-containing target genes. In these embryos, *nos*-driven *Gal4* is only expressed in the germline (future germ cells^28^) that give rise to the following generation. **b** Mating scheme of early embryonic Gal4 driver, *nos*-Gal4, to *UAS-bmm*. **c** Cumulative incidence of heart dysfunction and **d** TAG content in G1 adult flies after acute or parental exposure to NFD or HFD. *bmm* expression in the early embryo and future germ cells protected against HFD-associated heart dysfunction and fat accumulation. **e** Cumulative incidence of heart dysfunction and **f** TAG content in the second-generation progeny (G2) of parents (G0) fed a NFD or a HFD; and first-generation (G1) expressing *nos-*Gal4-driven ATGL/*bmm* in the germline. For heart dysfunction analysis *n* = 24–30 flies per condition (***p < 0.001) using the chi-square test (**c**, **d**). Statistical analysis of TAG content was conducted using one-way ANOVA, followed by Dunnett’s multiple comparisons test per genotype, *n* = 5–12 (**d**) and *n* = 2–10 samples per condition (**f**), *p < 0.05, **p < 0.01, ***p < 0.001 and ns not significant. Experiments were repeated at least twice with similar results. TAG content is expressed relative to control NFD-fed flies. Data represent mean ± SEM. Note that the second-generation progeny (G2) was protected against HFD-associated heart defects when the first progeny generation (G1) expresses *bmm* in the germline. *w*^*1118*^ flies were used as controls since they respond to HFD in a similar way compared to *UAS-Bmm*/*w*^*1118*^ and *nos-Gal4*/*w*^*1118*^ (Supplementary Figure 1). See Supplementary Figure 4 for driver specificity and additional heart parameters

We further tested the intergenerational metabolic reprogramming model by determining whether genetic manipulation of the very early embryo (G1) and the paternal germline of the next generation (G2) was sufficient to protect the adult G1 and G2 progeny from the adverse effects of a parental (or acute) HFD. For this purpose, we expressed *bmm* with a germline-specific driver, *nos*-Gal4, which resulted in *bmm* expression in the embryo at the mid-blastoderm stage (zygotic transcription start) through gastrulation as well as in the germ cells that give rise to the following generation^28^ (Fig. 5a, b and Supplementary Figure 4a, b). We found that *bmm* expression in the blastoderm embryo (G1) and its germline (future G2) protected G1 adult progeny from deleterious cardiac lipotoxicity due to parental exposure to a HFD (Fig. 5c; Supplementary Figure 1 and Fig. 4c-g). Importantly, this early embryonic G1 expression of *bmm* also conferred protection from HFD when the adult G1 flies were fed a HFD acutely (Fig. 5c, 6th and 8th bars). This suggests that early embryonic *bmm* expression protects the adult heart from both acute and parental HFD lipotoxicity. Next, male flies expressing *bmm* under the control of *nos-Gal4* driver were outcrossed to *w*^*1118*^ and fed a NFD or a HFD (Fig. 5b). Expression of *bmm* in the early embryo in G1 and the germ cells that become the G2 progeny conferred cardiac protection to acute or parental HFD in G2 flies (Fig. 5e and Supplementary Figure 4h-l). Similar re- and pre-programming effect was achieved when *bmm* expression was restricted to *Drosophila* adipose tissue during embryonic to adult stage in the offspring (G1) of HFD-fed parents, using *Lsp2-Gal4* driver. Expression of *bmm* during early development protected the following generation (G2) from the effects of parental or acute exposure to a HFD (Supplementary Figure 5a-d).

In line with these results, *bmm* expression using either *nos-Gal4* or *Lsp2*-Gal4 drivers both prevented HFD-induced fat accumulation in G1 and G2 flies (Figs. 4f, 5d, f and Supplementary Figure 5e). These data show that augmenting lipolysis in the early embryo or in adipose tissue is sufficient to *re-* or *pre-program* metabolism to shield the adult heart against parental, acute, or future HFD.

### Lipotoxic cardiomyopathy inheritance correlates with H3K27me3

**Fig. 6:**
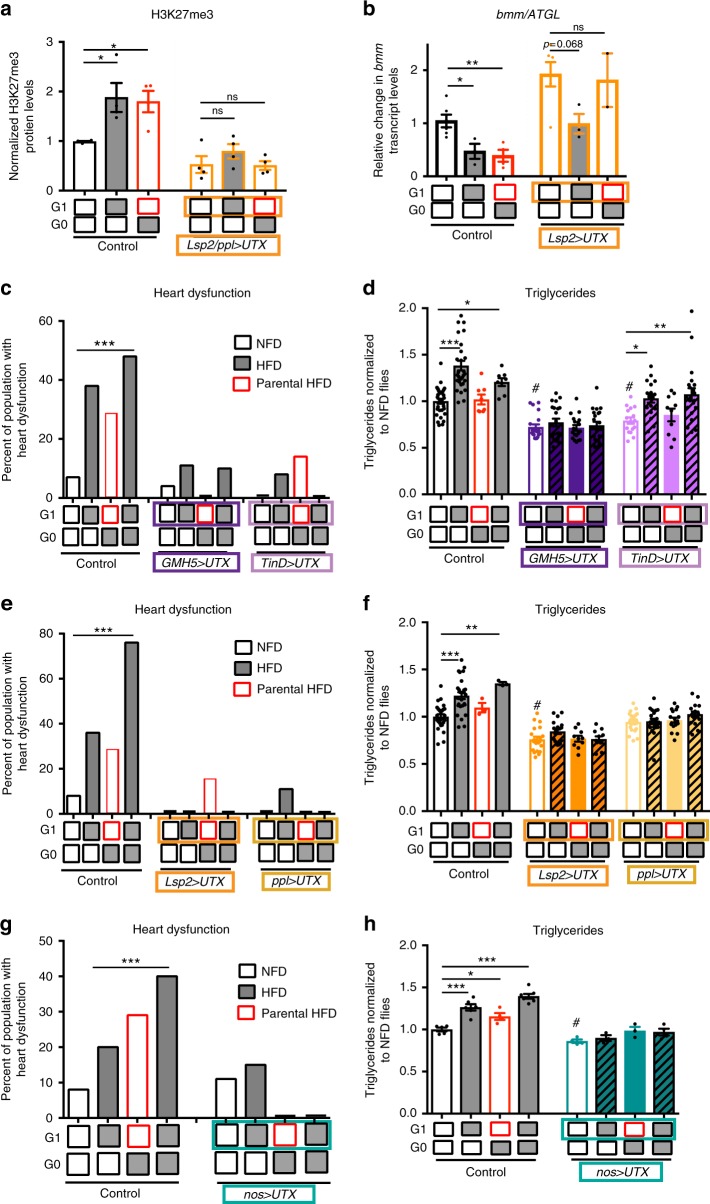
Overexpression of the demethylase UTX reduces H3K27 trimethylation and protects against the effects of parental and acute exposure to a HFD. **a** Quantification of H3K27me3 protein abundance by western blot analysis in G1 whole flies after acute or parental exposure to NFD or HFD. Bars show quantification of H3K27me3 normalized to Histone 3 (H3) levels. Data are the means ± SEM of 4 independent experiments (*p < 0.05 and ns not significant, compared to NFD control flies, one-way ANOVA, followed by Dunnett’s multiple comparison test per genotype). Control flies showed a significant HFD-associated increase in systemic H3K27me3 protein levels. Adipose-restricted (*Lsp2*/*ppl*>Gal4 driven) expression of the H3K27me3-specific demethylase dUTX reduced H3K27me3 levels independently of the dietary conditions. See Supplementary Figure 8a for representative blots. **b** *bmm* transcript levels in control flies are decreased by acute or parental exposure to a HFD. Expression of *dUTX* prevents *bmm* transcript levels reduction under all dietary conditions. Results are normalized to *rp49* expression and expressed relative to control NFD (*n* = 2–6 samples, *p < 0.05, ** *p* < 0.01 and ns not significant vs. NFD, one-way ANOVA, followed by Dunnett’s multiple comparisons test per genotype). **c**, **d** Cumulative incidence of heart defects (**c**) and TAG accumulation (**d**) in G1 flies. Expression of *dUTX* in either myocardial progenitor cells (*TinD*-Gal4) or adult myocardial cells (*GMH5*-Gal4) protected against the adverse effects of both acute and parental HFD exposure. *dUTX* overexpression in adult myocardium protected against excess TAG accumulation upon acute HFD but expression in the embryonic heart progenitors was not protective. **e**, **f** Adipose-specific expression of *dUTX* driven by either *ppl*-Gal4 or *Lsp2*-Gal4 protected against acute and parental HFD-associated heart dysfunction (**e**) and HFD-induced TAG accumulation (**f**). *nos*-Gal4-driven *dUTX* expression in the early embryonic tissue protected against acute and parental HFD-associated heart dysfunction (**g**) and TAG accumulation (**h**). In (**c**), (**e**) and (**g**) heart dysfunction was analyzed by the chi-square test, *n* = 25–30 per condition. In (**d**), (**f**), and (**h**), TAG content is normalized to control flies fed NFD in G0 and G1. Each genotype was analyzed by one-way ANOVA, followed by Dunnett’s multiple comparisons test *p < 0.05, **p < 0.01, ***p < 0.001 vs NFD of its same genotype and ^#^p < 0.05 compared to control NFD (*n* = 8–30 (**d**), *n* = 3–27 (**f**), and *n* = 3–7 (**h**) samples each one consisting on 3 pooled flies per condition). Mean ± SEM are presented. A mild increase in TAG content was observed upon parental HFD feeding in control flies (**h**). *w*^*1118*^ flies were used as control. See Supplementary Figures 6 and 7 for additional heart parameters

Changes in gene expression could be transmitted from one generation to the next through inheritance of epigenetic alterations^29–32^. Given the emerging relationship between nutrient availability and epigenetic regulation of gene expression^9,33,34^, we sought to investigate the relationship between intergenerational lipotoxic cardiomyopathy inheritance, reduced expression levels of *ATGL/bmm* and *PGC-1*, and epigenetic repression of gene expression. In *Drosophila*, maternal HFD and HSD are known to modify the expression of metabolic genes in adult offspring^18,35^ and paternal HSD can modify the offspring’s chromatin state and gene transcription in a H3K9/K27me3-dependent manner^19^. In relation to heart function, *Ubiquitously transcribed tetratricopetide repeat gene on the X chromosome* (*UTX*), a highly conserved H3K27 demethylase, has been shown to promote a cardiac-specific gene program^36^, while the catalytic subunit of the PRC2 complex, the H3K27 methyltransferase EzH2, is required to stabilize postnatal cardiac gene expression by promoting gene repression^37^. Thus, we first monitored global levels of H3K27me3 to explore the involvement of the H3K27me3 repressive marks in the intergenerational inheritance of lipotoxic cardiomyopathy. Indeed, acute exposure of adult flies to a HFD caused a significant increase in whole-body H3K27me3 levels that persisted in the next NFD-fed generation (Fig. 6a and Supplementary Figure 8a).

To determine whether H3K27me3 is a key epigenetic regulator of intergenerational HFD-related pathology, we reduced H3K27me3 levels by overexpressing *UTX*. Adipose-restricted expression of *Drosophila UTX* (*dUTX*^38^) using *Lsp2*-Gal4 not only prevented the HFD-induced increase in H3K27me3 levels but also significantly increased *bmm* messenger RNA (mRNA) levels, even under acute or parental HFD conditions (Fig. 6a, b). Interestingly, the elevated levels of *bmm* expression due to *dUTX* overexpression in animals fed NFD are mildly reduced by an acute HFD but not a parental HFD (Fig. 6b, right panels). Therefore, acute exposure to a HFD may not lower *bmm* transcription solely by regulating H3K27me3 levels. However, it is important to note that *bmm* transcript levels in HFD-fed *dUTX* expressing flies were similar to the levels in NFD-fed control flies (Fig. 6b).

In order to study the effect of manipulating H3K27me3 levels on heart function, we next used heart-specific drivers to overexpress *UTX*. Cardiac-specific (*GMH5-Gal4*) or cardiac progenitor-specific (*TinD-Gal4*) *dUTX* expression effectively protected against cardiomyopathies due to acute or parental HFD exposure (Fig. 6c and Supplementary Figure 6a-e), as was observed by overexpressing *bmm* using these same drivers. It should be noted that *dUTX* expression in the embryonic and adult myocardium, but not in cardiac progenitors, prevented systemic fat accumulation upon acute HFD feeding (Fig. 6d). This contrasts with GMH5-driven *bmm* expression, which had no effect on HFD-induced systemic fat accumulation (Fig. 4c). Taken together, these findings suggest that genetic manipulations in adult myocardial cells differ in their potential to affect systemic metabolism. Moreover, *Lsp*2-Gal4- and *ppl-Gal4*-driven adipose-restricted expression of *dUTX* also provided protection against HFD-associated heart dysfunction and HFD-induced fat accumulation across generations (Fig. 6e, f and Supplementary Figure 6f-j). In line with an increase in *bmm* transcript levels, *Lsp2*>*UTX* flies presented a mild reduction in fat content in NFD conditions when compared to NFD control flies.

Finally, we found that *nos*-Gal4-driven expression of *dUTX* specifically in the early blastoderm embryo (G1) and future germline (G2) preserved all aspects of heart function and prevented excess fat accumulation following acute or parental exposure to a HFD (Fig. 6g, h and Supplementary Figure 7), similar to observations in animals with *nos-*Gal4-driven overexpression of *bmm* (Fig. 5c–i). These data support the conclusion that *dUTX* overexpression and thus reduction of H3K27me3 levels, as early as in the germline, has a profound effect on metabolic state and cardiac function later in life.

### EzH2 inhibition prevents HFD-induced lipotoxic cardiomyopathy

**Fig. 7:**
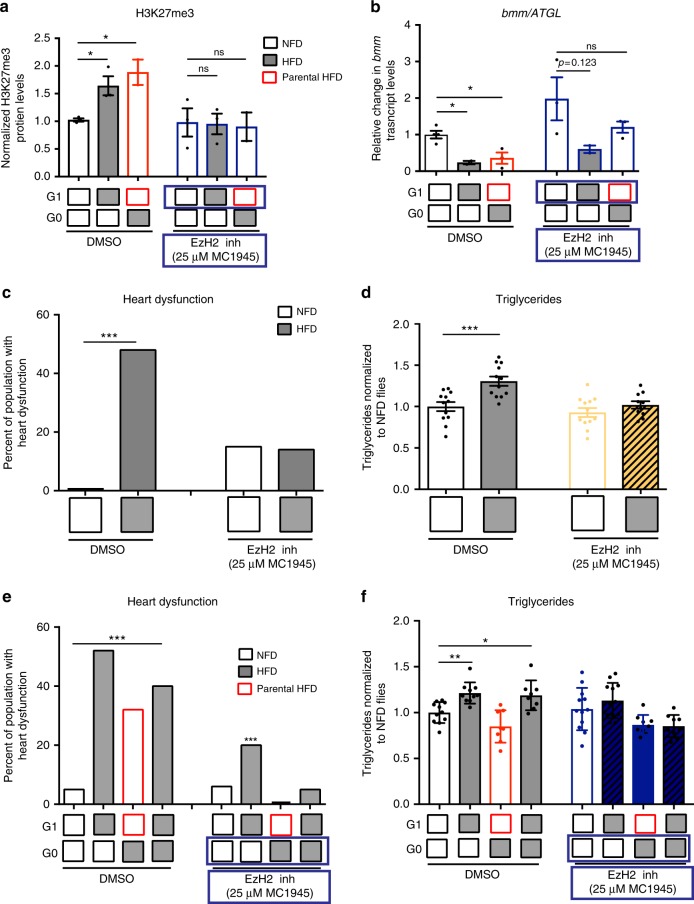
Pharmacological inhibition of PRC2 component EzH2 reduces H3K27 trimethylation and protects against the effects of parental and acute exposure to HFD. **a** Western blot analysis and quantitative densitometry of H3K27me3 protein levels, normalized to H3 in whole G1 adult flies after acute or parental exposure to NFD or HFD. Data are the means ± SEM from at least 2 independent experiments, *n* = 2–3 samples per condition. Inhibition of EzH2 activity prevented any HFD-induced H3K27me3 content increase, similar to the effect of d*UTX* overexpression (Fig. 6a). See Supplementary Figure 8a for representative blots. **b** *bmm* transcript levels were decreased by acute or parental exposure to HFD, in control DMSO-fed flies, whereas EzH2 inhibition prevented any significant effect on transcript levels under all dietary conditions, *n* = 2–4 samples per condition. Results in (**a**, **b**) are normalized to DMSO-treated flies fed NFD in G0 and G1 (*p < 0.05, **p < 0.01, ***p < 0.001 vs NFD analyzed by one-way ANOVA, followed by Dunnett’s multiple comparisons test per genotype). **c**, **d** Cumulative incidence of heart defects (**c**) and TAG content (**d**) in flies treated with 25 μM EzH2 inhibitor (MC1945) or DMSO exposed to a NFD or a HFD. Treatment with EzH2 inhibitor protected against the adverse effects of acute HFD on heart function (**c**) and fat accumulation (**d**). **e**, **f** Supplementation of the parental diet (G0) with the EzH2 inhibitor protected the progeny against the adverse effects of acute or parental exposure to a HFD. The progeny of parents fed the EzH2 inhibitor was protected against heart dysfunction (**e**) and excess TAG accumulation (**f**) due to acute or parental exposure to HFD. Statistical analysis of heart dysfunction was conducted with chi-square test, *n* = 25 flies. Statistical analysis of TAG content was conducted using one-way ANOVA, followed by Dunnett’s multiples test per genotype, *n* = 8–12 samples per condition. In (**e**, **f**), TAG content data are expressed as mean ± SEM and normalized to control flies fed NFD in G0 and G1. These experiments were repeated at least twice with similar results. ***p < 0.001, **p < 0.01, and *p < 0.05 were considered statistically significant vs NFD from same genotype

Since inhibition of H3K27me3 by *dUTX* overexpression protects the fly from the deleterious effects of both acute and parental HFD exposure, we also examined whether reducing H3K27me3 levels pharmacologically, by feeding the flies with an inhibitor of the H3K27 methyltransferase subunit EzH2 of the PRC2, is also protective^39^. We found that exposure of adult flies to the EzH2 inhibitor, MC1945, prevented the global increase in H3K27me3 levels observed upon acute and parental HFD conditions in control dimethyl sulfoxide (DMSO)-treated flies (Fig. 7a, and Supplementary Figure 8a). Importantly, flies exposed to MC1945 exhibited an increase in *bmm* expression under NFD and no significant reduction in transcript levels upon acute or parental HFD (Fig. 7b), which is strikingly similar to the phenotype induced by adipose-restricted *dUTX* expression (compare to Fig. 6b). Notably, pharmacological inhibition of H3K27me3 production with 25 μM of MC1945 (selected from dose–response studies; Supplementary Figure 8b-e) had no detectable effect on cardiac function or TAG content of NFD-fed flies, but significantly reduced the percentage of flies with heart dysfunctions triggered by an acute HFD and fully protected the flies from fat accumulation upon HFD exposure (Fig. 7c, d). Next, we collected the progeny from flies that had been exposed to HFD or NFD containing EzH2 inhibitor (or DMSO). As expected, EzH2 inhibitor (in G0) also protected the offspring from the detrimental effects of parental exposure to a HFD on heart function (Fig. 7e and Supplementary Figure 8f-j). Remarkably, however, such parental EzH2 inhibition also reduced or eliminated heart dysfunctions when their progeny were exposed to an acute HFD (in G1) (Fig. 7e and Supplementary Figure 8f-j). In line with these results, parental exposure to EzH2 inhibitor also prevented triglyceride accumulation in the progeny that was fed an acute HFD (Fig. 7f). While further experiments are required to determine the direct regulation of *bmm* transcript levels and the specific gene targets of EzH2 and *dUTX*, we provide multiple lines of evidence to demonstrate that exposure to HFD can elevate H3K27me3 across the genome and that this effect persists into the next generation, thereby mediating the inheritance of lipotoxic cardiomyopathy.

## Discussion

Several studies have established a critical role for TAG hydrolysis in cardiac metabolism and function, in both healthy and diseased hearts^21,40,41^. Here we show that acute HFD (5 days of food supplemented with 30% coconut oil) induces lipotoxic cardiomyopathy that can be inherited by the next two generations, via the parental germlines, even when the offspring are raised on NFD. Similar to what has been described by epidemiological studies on the offspring of obese pregnant women^42^, we found in our fly model that parental HFD led to first generation progeny with increased adult body weight and increased fat content in late-stage embryos. This was no longer the case in second generation offspring, and adult progeny did not exhibit an increase in fat content relative to body weight in either generations. In contrast, metabolic reprogramming across generations was particularly evident in the systemic reduction in the transcript levels of *ATGL/bmm* lipase and its downstream target *PGC-1/spargel*, a key regulator of energy metabolism. We further validated the intergenerational lipotoxic cardiomyopathy model by genetically reducing *PGC-1* expression in the parents (*PGC-1*^*xp*^ heterozygotes), which causes similar cardiac lipotoxicity as HFD exposure^16^. This was sufficient to alter the +/+ offspring’s metabolic state, leading to lipotoxic cardiomyopathy later in life, even though these flies carry two wild-type copies of *PGC-1*. Of note, the partial reduction of *PGC-1* induced by acute and parental HFD in control flies and *PGC-1*^*XP*^ heterozygous mutant flies is likely having a profound effect on mitochondrial biogenesis that could be underlying the observed heart dysfunction.

The experiments presented here indicate that HFD and reduced *PGC-1* expression levels have the ability to modify the offspring’s metabolism leading to heart dysfunction. Thus, we speculated that parental HFD-dependent metabolic reprogramming and associated lipotoxic cardiomyopathy in the progeny could be prevented by increasing ATGL/*bmm* levels. Indeed, targeted transgenic expression of *bmm* is able to reset the altered metabolic state induced by parental HFD, and thus protects the progeny from cardiac lipotoxicity. Remarkably, induction of *bmm* expression in the early embryo is sufficient to render the adult progeny (G1), as well as the following generation (G2), resistant to acute HFD.

The presented data support the hypothesis, first formulated by Barker^43^, that the quality of nutrition during gestation leads to fetal programming that functions as a key determinant in establishing predisposition and/or susceptibility to metabolic and cardiovascular disorders later in life. While this hypothesis is sustained by several epidemiological studies, including the Dutch Hunger Winter studies^44^, and many animal models with different environmental stressors^45^, the underling mechanisms on the intergenerational inheritance of lipotoxic cardiomyopathy induced by parental HFD remain mostly unknown. The *Drosophila* model established here indicates that the inheritance of altered histone modifications is a key mechanism in the propagation of lipotoxic cardiomyopathy across generations.

Histone modifications are essential in regulating chromatin packaging and gene expression for proper development and cell function. In turn, metabolites serve as co-factors for chromatin modifying enzymes, allowing protein activity and gene expression to match the specific energy requirements^41^. We found that HFD, and potentially its maladaptive fluctuation in metabolites, can lead to changes in chromatin structure and gene expression that are transmitted to the next generation. Indeed, a major finding presented here is the involvement of the PRC2 complex in regulating metabolism and heart function in response to a HFD, via H3K27me3 gene repression across generations. The increase in H3K27me3 global levels in the adult flies upon acute or parental HFD supports the hypothesis that HFD causes changes in the epigenome that have lifelong consequences. Our results are in line with previous findings in *Caenorhabditis* *elegans* and mice, which provide evidence that PRC2-mediated epigenetic modifications in the germ cells can be transmitted to embryos by sperm and/or oocytes^46,47^. In addition, we found that reduction of H3K27me3, either by overexpression of UTX or inhibition of EzH2, can prevent the deleterious effects of a parental HFD on heart function and metabolism. Of note, at this point we cannot rule out that UTX overexpression and EzH2 inhibition might also have H3K27me3-independent effects.

Overall, using the genetic model of *Drosophila*, we provide first evidence that early embryonic and tissue-specific modulation of lipolysis in myocardial progenitors, adipose tissue, and the germline leads to tissue-specific and/or systemic metabolic reprogramming or pre-programming that persists into adulthood. Importantly, the imposed metabolic state appears to be inherited by the next two generations as either a predisposition to metabolic imbalance and cardiac dysfunction, or a protection from HFD insult when *bmm* is overexpressed. Moreover, we provide evidence that metabolic re-programming that leads to fat accumulation and cardiac lipotoxicity correlates with overall levels of H3K27me3 epigenetic marks, which can be reversed by genetic or pharmacological reduction of H3K27me3 levels. Our findings shed light on possible causes of obesogenic heritability and early adult onset of cardiovascular disease and diabetes, which appear to have their roots in the diet and overall metabolic state of the parents. Importantly, we demonstrate that targeted genetic or pharmacological interventions in the progeny are able to counteract the detrimental effects on cardiac function of parental dietary insults, a protection that persists even in the subsequent generation against acute HFD. These findings provide new perspectives for tackling metabolic syndrome effects across generations and preventing lipotoxic cardiomyopathies.

## Methods

### Fly stocks

We obtained *w*^*1118*^ flies (used as controls in Figs. 3–7) from the Bloomington Drosophila Stock Center. We used multiple drivers for each tissue type. For expression in the fat body we used *Lsp2*-Gal4^26^ and *ppl*-Gal4^27^; for the heart, we used *GMH5*-Gal4^48^ and *TinD*-Gal4^25^. The UAS-*bmm* overexpression line was provided by Ronald P. Kühnlein (Max-Plank Institute)^49^, *nos*-Gal4 flies^28^ were a gift from R. Zhou (Sanford Burnham Prebys Medical Discovery Institute), and the UAS-*dUTX*^38^ flies were provided by A. Bergmann (University of Massachusetts Medical School). *PGC-1*^*XP*^ (CG9809^d04518^) flies were obtained from Harvard Medical School. All flies were maintained on a NFD composed of yeast, corn starch, and molasses (10% yeast, 12% sugar, and 1.5% agar)^50^. The HFD was made by mixing 30% (weight/volume) coconut oil with NFD. This formulation was used for all experiments because it gave the strongest phenotype and the most reproducible results^15^.

### Intergenerational HFD and NFD feeding regimen

Male and female flies were collected after eclosion and aged for 5 days on NFD (40 flies per tube). Flies were then randomly placed on NFD or HFD for an additional 5 days. Each population was then placed on NFD (see schematic in Fig. 1a) and allowed to lay eggs for 3 days. The offspring were raised on NFD. After the flies emerged (following pupation), they were aged for 5 days on NFD, and then randomly placed on NFD or HFD for 5 more days. Next, mated females were used to assess heart function, to measure TAG levels, and to extract RNA. Additionally, 20 females and 20 males were placed on NFD and allowed to lay eggs for 3 days to create the subsequent generation. This protocol was repeated for 3 generations (see Fig. 1a). Flies were housed in a 21 °C incubator with 50% humidity and controlled 12 h light/dark cycle. Embryos were collected on agar plates following standard protocols^51^. In brief, flies were placed in bottles containing an agar plate with 10% concentrated grape juice on the bottom and incubated at 25 °C for the specific time lapses described (2 or 13 h). Embryos were then recovered from the agar plates and processed for TAG assessment or RNA extraction. For the EzH2 inhibition treatment, we ran a dose–response test in order to find the best concentration (see Supplementary Figure 8b-e). Then, 100 μl of DMSO (vehicle) or 25 μM EzH2 inhibitor (MC1945) was added to the surface of the vials containing NFD or HFD standard food.

### Heart preparation and analysis

Surgical dissection and exposure of hearts, high-resolution video microscopy, and analysis of movies for cardiac function measurements were performed according to published procedures^52^. Female flies were dissected in oxygenated artificial hemolymph in order to expose their hearts within the abdomen. High-speed digital movies were recorded using HCImageLive software (Hamamatsu) and then analyzed in a group-blinded fashion for heart rate, arrhythmia, contractility, and fractional shortening using SOHA software^52^. The following formula was used to determine sample size for a significance level of 5% with a power of 90%: *n* *=* 1 + 21 × (standard deviation/*d*)^2^, where *d* = size of difference in means^53^. For most of the experiments, a sample size of 15–20 flies was adequate.

### Triglyceride measurement

TAGs were measured as described by Diop et al.^50^. In brief, whole female flies were placed in empty vials for 30 minutes (min), then transferred to Eppendorf tubes in batches of 12, weighed, and either processed immediately or stored at −80 °C for later processing. Flies were pooled in groups of 3 and homogenized in 600 µl of phosphate-buffered saline (PBS) containing 0.05%Triton X-100 for 5 min at 10,000 rpm using a High Throughput ball bearing Homogenizer (Talboys). The homogenates were centrifuged at 3000 × *g* for 15 min, and 20 µl of the supernatant was transferred into 200 µl of Triglyceride Reagent (Thermo Electron Corp. catalog number TR22421/2780-250). The reaction mixture was placed on a shaker at 300 rpm and incubated at 37 °C for 10 min. The absorbance at 550 nm was then measured using SpectraMax M2e (Molecular Devices Corp.) and TAG content was calculated by comparison with a standard curve constructed with solutions of known concentrations of TAG (Pointe Scientific, catalog number T7531-STD). The Triglyceride Reagent contains a Lipoprotein lipase that hydrolyzes the triglycerides present in the sample and produces glycerol, which is processed by a series of reactions ultimately producing a red-colored dye whose absorbance is proportional to the concentration of triglycerides present in the sample^54^. The protein content of the same samples was measured using Quick Start Bradford reagent (Bio-Rad). TAG levels are expressed as TAG/protein for embryos and TAG/fly weight for adults and normalized to *w*^*1118*^ NFD flies.

### Immunohistochemistry

The 1–2-week-old female flies were dissected in oxygenated artificial hemolymph to expose the heart and surrounding fat tissue. Samples were fixed for 20 min in 4% paraformaldehyde diluted in PBS, washed 3 times in PBS containing 0.3% Triton (PBS-T), and incubated overnight at 4 °C with the primary antibodies diluted in PBS-T/anti-GFP-1020^55^, 1:1000 (Aves lab), and anti-α-spectrin (3A9)^56^, 1:50 (DSHB). Tissues were washed 3 times in PBS-T and incubated for 2 h at room temperature with secondary antibodies: Alexa Fluor 647 and Alexa Fluor 555, 1:500 (Invitrogen), and 4′,6-diamidino-2-phenylindole (DAPI; 0.5 µg ml^−1^, Invitrogen) in PBS-T. After 3 washes with PBS-T, samples were placed in PBS and subsequently mounted onto glass slides using Prolonged Gold (Invitrogen) as the mounting media. Images were acquired on a Zeiss AxioImager Z1 equipped with an Apotome (Carl Zeiss) and an ORCA-Flash 4.0LT Digital CMSO camera C11440 (Hamamatsu), using Zen 2.3 pro software. Images were analyzed using ImageJ 1.49 m software.

### Real-time quantitative PCR (qPCR) analysis

To check the transcriptional expression of *bmm* and *PGC-1* in adult flies, ~12 whole female flies were flash-frozen in liquid nitrogen and homogenized in 700 μl of ice-cold QIAzol Lysis Reagent (Qiagen) using a pellet pestle motor (Kontes) according to the manufacturer’s instructions. To check the transcriptional expression of *bmm* in embryos, these were recovered at specified time points from agar plates with a brush and dechorionation was performed using 50% bleach^57^. Next, embryos were placed into Eppendorf tubes containing ice-cold PBS-T and centrifuged for 2 min at 3300 × *g*. The PBS-T was then completely removed, and embryos were re-suspended in 300 μl of ice-cold QIAzol Lysis Reagent (Qiagen). Total RNA was extracted from adult whole-body or embryos homogenates using the miRNeasy kit (Qiagen). Then, 500 ng of RNA was used to synthesize complementary DNA with the QuantiTect Reverse Transcription (RT) Kit (Qiagen). RT-qPCR was performed according to the manufacturer’s protocol using FastStart Essential DNA Green Master and a LightCycler® 96 System (ROCHE). mRNA levels were calculated relative to *w*^*1118*^ NFD control flies using the ΔΔCt method. *rp49* was used as an internal control to normalize the total amount of RNA. Primer sequences were as follows:

*PGC-1* F: 5’-CCATTGACACGAGTCATAGAGAC-3’;

*PGC-1* R: 5’-AGCATTATCCAAGTCAGCGTAA-3’;

*rp49* F: 5’-GCTAAGCTGTCGCACAAATG-3’;

*rp49* R: 5’- GTTCGATCCGTAACCGATGT-3’;

*bmmA* F: 5’-CCTTCACCCACTCAAGCATT-3’;

*bmmA* R: 5’-GCCGTGGAGCTAAAAGTCTG-3’.

### RNA in situ hybridization (RNAscope®)

Probes against *Gal4* were generated using RNAscope® (ACD) platform. Female flies were dissected for RNA in situ hybridization as previously described^58^ and the manufacture’s protocol was followed. *Gal4*-labeled hearts were mounted immediately after using Prolong Gold (Molecular Probes). Images were acquired using an Apotome.2 (Zeiss), ZEN 2.3 pro software and later analyzed using ImageJ.

### Histone extraction and western blot analysis

Approximately 40 flies per sample were pulverized in liquid nitrogen, and histones were extracted. Briefly, each sample was suspended in Triton Extraction Buffer (TEB: PBS containing 0.5% Triton X-100, 2 mM PMSF, and 0.02% NaN3) and incubated on ice for 10 min. During this incubation, samples were vortex mixed every 3 min. Lysates were centrifuged at 6500 × *g* for 10 min at 4 °C. The formed pellets were washed with TEB, vortex mixed and centrifuged at 6500 × *g* for 10 min at 4 °C. The recovered pellet was then re-suspended in 0.2 N HCl overnight at 4 °C. Samples were centrifuged at 6500 × *g* for 10 min at 4 °C and the supernatant was transferred to a clean tube. The protein concentration of each sample was determined with a Micro BCA Protein Assay Kit (ThermoScientific). About 40 μg of protein per sample was resolved by sodium dodecyl sulfate–polyacrylamide gel electrophoresis, transferred to nitrocellulose membranes, and incubated overnight at 4 °C with anti-H3K27me3^38^ (1:1000, Active Motif, catalog number 39155) or anti-H3 (1:1000, Millipore, catalog number 06-755) antibodies in Tris-buffered saline, 0.05% Tween 20 (TBS-T), containing 5% of bovine serum albumin (BSA). After 3 washes of 10 min with TBS-T, blots were incubated for 1 h at room temperature with horseradish peroxidase-conjugated anti-rabbit secondary antibody (Amersham) diluted 1:5000 in TBS-T containing 5% of BSA. Blots were washed again and bands were visualized using enhanced chemiluminescent reagent (ECL Prime Western Blotting Detection Reagent, Amersham). Densitometry quantification was achieved using ImageJ software.

### Statistical analysis

Statistical analyses of TAG, mRNA levels, and H3K27me3 content was performed using one-way analysis of variance (ANOVA) and Dunnett’s multiple comparisons test against NFD among same genotypes or Sidak’s multiple comparisons test when different genotypes but same conditions were compared. Heart function measurements were analyzed using unpaired, two-tailed, Student’s *t*-test (for two group comparison) or one-way ANOVA followed by Sidak’s multiple comparisons test within same genotypes. Heart dysfunction phenotypes (partial conduction block, non-contractile cells in the heart, ostia defects) were analyzed using the chi-square test and one representative experiment is shown. Flies were assigned a numerical code per genotype/treatment so investigators were blinded when analyzing heart function. Outliers were identified using the ROUT method, *Q* = 1%. Statistical analysis was performed using GraphPad Prism (GraphPad Software, La Jolla, California, www.graphpad.com). Values are presented as means ± SEM. The *p* values < 0.05 were considered significant. All data presented here are representative of at least two independent experiments.

### Reporting summary

Further information on experimental design is available in the Nature Research Reporting Summary linked to this article.

## Supplementary information

Supplementary Info

Reporting Summary

## Data Availability

The authors declare that the data supporting the findings of this study are available in the article and its Supplementary Information File. All other relevant data supporting the findings of this study are available from the corresponding author upon reasonable request.
