# Supplementary material for: Intergenerational inheritance of high fat diet-induced cardiac lipotoxicity in Drosophila
Source: Nat Commun. 2019 Jan 14;10:193. doi: 10.1038/s41467-018-08128-3 (PMC6331650; doi:10.1038/s41467-018-08128-3)
Supplement: Supplementary file 1 — Supplementary Info [file 41467_2018_8128_MOESM1_ESM.pdf]

Supplementary Information

Intergenerational inheritance of high fat diet-induced cardiac lipotoxicity in *Drosophila*

Guida, Birse et al.

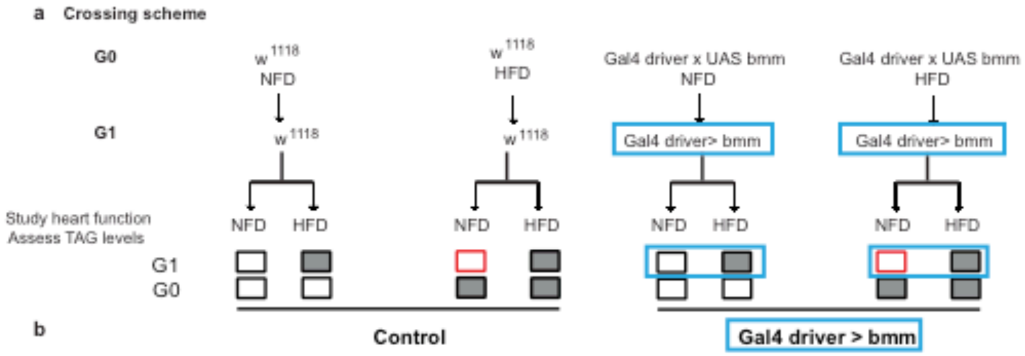

b

		N	Diastolic Diameter average	SEM	T-test	Systolic Diameter average	SEM	T-test	Fractional Shortening average	SEM	T-test
UAS *bmm* x *w1118*	HFD	22	66.05	1.82		42.45	1.79		0.35	0.02	
UAS *bmm* x *w1118*	ND	22	78.63	1.83	p<0.0001	46.19	1.05	p>0.05	0.41	0.02	p<0.05
TinD-Gal4 x *w1118*	HFD	11	63.17	3.12		44.78	2.69		0.29	0.02	
TinD-Gal4 x *w1118*	ND	14	76.91	2.36	p<0.01	50.27	2.49	p>0.05	0.35	0.01	p<0.05
GMH5-Gal4 x *w1118*	HFD	18	59.11	2.90		37.07	2.08		0.37	0.02	
GMH5-Gal4 x *w1118*	ND	18	84.45	3.09	p<0.0001	51.92	2.22	p<0.0001	0.38	0.02	p>0.05
Lsp2-Gal4 x *w1118*	HFD	15	68.72	2.08		39.62	1.64		0.42	0.01	
Lsp2-Gal4 x *w1118*	ND	13	77.50	2.01	p<0.01	43.78	1.52	p>0.05	0.43	0.01	p>0.05
ppl-Gal4 x *w1118*	HFD	13	62.38	3.67		38.94	1.73		0.37	0.02	
ppl-Gal4 x *w1118*	ND	12	73.71	2.43	p<0.05	42.39	1.97	p>0.05	0.43	0.10	p<0.05
nos-Gal4 x *w1118*	HFD	11	58.47	2.11		38.39	1.62		0.34	0.01	
nos-Gal4 x *w1118*	ND	13	80.97	3.10	p<0.0001	50.63	2.73	p<0.01	0.38	0.02	p<0.01
w1118	HFD	23	64.21	1.90		40.67	1.04		0.34	0.01	
w1118	ND	22	71.23	2.04	p<0.05	44.67	1.59	p<0.05	0.39	0.01	p<0.01

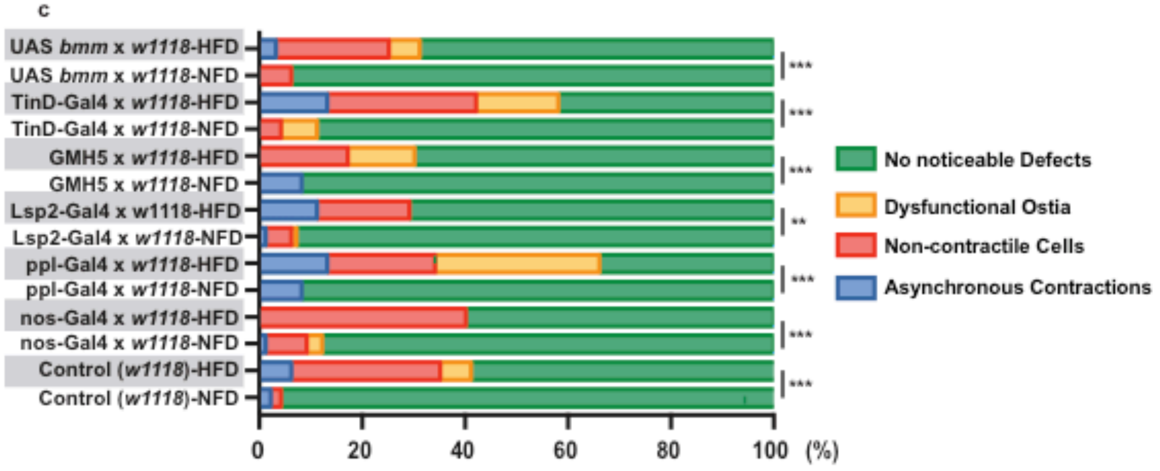

Supplementary Figure 1. Experimental set up of an intergenerational model of HFD-induced lipotoxic cardiomyopathy in *Drosophila*. (a) Graphical representation of the crossing scheme for Figs.4-6. Specific Gal4 drivers were crossed to UAS *bmm* virgins (or UAS *dUTX* in Fig. 6) and kept for 5 days on a normal food diet (NFD) or a high-fat diet (HFD). Flies were transferred to NFD and allowed to lay eggs for 3 days. After eclosion, the progeny (G1) was collected and placed on vials with NFD with 30 flies per vial, for 5 days. Next, flies were fed a NFD or a HFD for 5 days. Subsequently after dietary exposure flies were used to study heart function, RNA extraction or assess TAG levels. Blue box depicts the expression of UAS *bmm* (or UAS *dUTX* in Fig 6). w^{1118} flies were put in parallel on the same regimen and used as controls. Grey boxes symbolize HFD feeding and red

boxes parental-HFD feeding. (b) Table showing the control crosses performed to validate the drivers and UAS constructs used in this study. All lines showed a statistically significant decreased in diastolic diameter as expected. Fractional shortening was reduced in all lines but not statistically significant reduced in *GMH5*-Gal4 and *Lsp2*-Gal4 suggesting that these two lines are less sensitive to dietary insult for these parameters. (c) Cumulative incidence of heart dysfunctions, including ostia defects, non-contractile and partial conduction block, were measured in the Gal4 driver lines crossed to w^{1118} flies as well as UAS *bmm* line crossed to w^{1118}. Flies were exposed to 5 days of HFD or NFD. HFD causes significant increase in occurrence of heart dysfunctions (Chi-square test, ****p<0.0001, N=15-23 flies per genotype). The experiments were repeated at least two times with similar results.

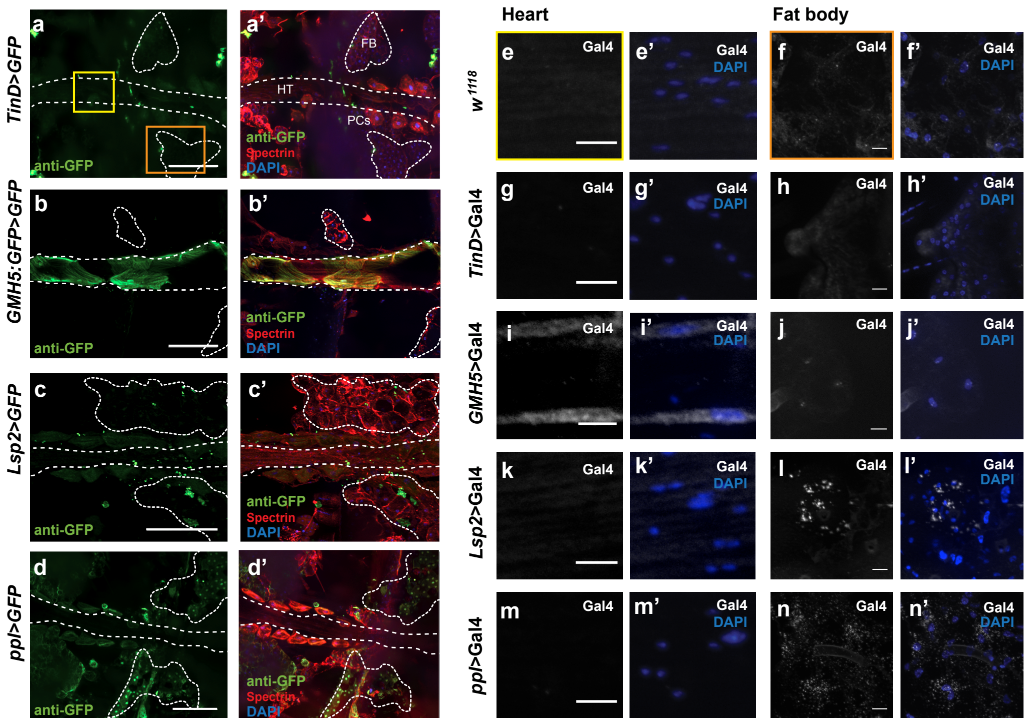

Supplementary Figure 2. Validation of Gal4 drivers. (a-d) Anterior view of *Drosophila* adult heart and surrounding Fat Body. Heart tube (HT) and Fat Body (FB) are delimitated by a dotted line. Pericardial cells (PCs) can be observed aligned to both sides of the HT. Expression of GFP was detected by antibody staining and an antibody against the cytoskeletal plasma membrane associated protein, Spectrin, was used to mark the edges of the cells (anti-GFP, green; anti-alpha-Spectrin, red) in 10 days-old flies. (a) *TinD-Gal4* showed no expression of GFP in the HT or the FB in adult flies. (b) Expression of GFP in the HT, but not the FB, was detected when using *GMH5-Gal4* driver, (c, d) *Lsp2-Gal4* and *ppl-Gal4* showed expression of GFP in the FB but not in the HT. Note that *ppl-Gal4* induced stronger expression of GFP at this time point compared to *Lsp2-Gal4*. *ppl-Gal4* also showed mild expression of GFP in PCs. Scale bars are 50μm. (e-m) Hearts were dissected, fixed and incubated with *Gal4* specific probes for RNA hybridization. The hybridization signal was amplified by RNAscope® detection reagents and the punctate dot signal, representing the single Gal4 RNA molecules, were visualized under the microscope. A yellow and orange square in (a) illustrates the specific regions of the heart and the Fat Body respectively, that were used for the RNAscope® analysis. (g-j) *TinD-Gal4* showed no expression of *Gal4* in adult cardiomyocytes nor in adipose tissue while, *GMH5*-Gal4 showed strong signal in the cardiomyocytes and no expression of *Gal4* RNA in adult adipose tissue. (k-n) *Lsp2-Gal4* and *ppl-Gal4* both showed specific *Gal4* transcripts in adipose tissue, while *Gal4* messages were not detectable in cardiomyocytes. (e, f) w^{1118} was used as a negative control. No *Gal4* messages were

detected in the heart or the fat body of w^{1118} flies. Scale bars are 10μm. Images are representative of more than two independent experiments.

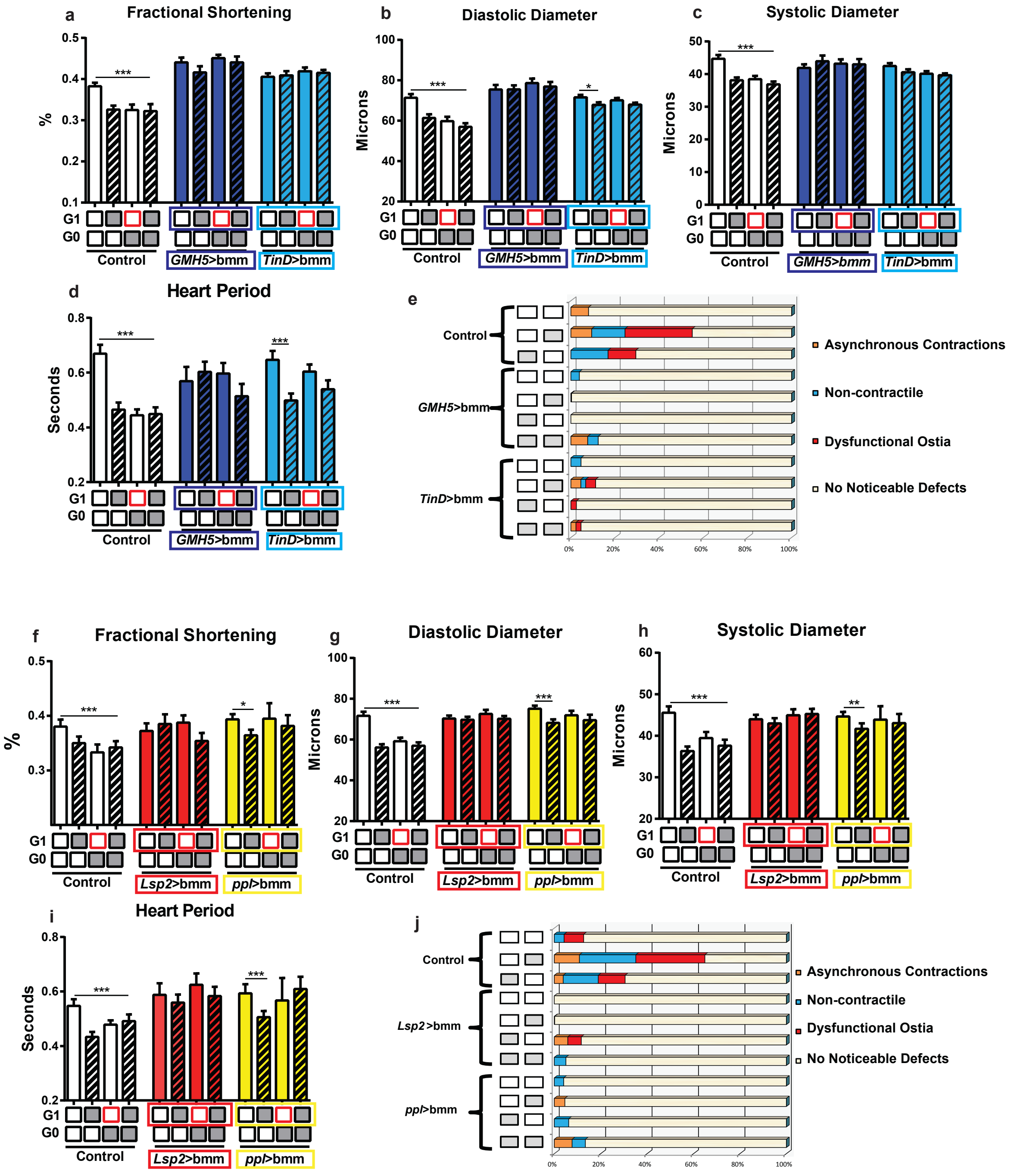

Supplementary Figure 3. Expression of *bmm* in cardiac and adipose tissue protects from adverse effects of HFD and parental HFD on heart function. (a–d) Fractional shortening (a), diastolic diameter (b), systolic diameter (c), and heart period (d) in control

G1 flies are significantly decreased by acute or parental exposure to a HFD. Expression of *bmm* in adult myocardial cells (*GMH5*-Gal4>UAS-bmm) protected against the adverse effects of a HFD on all heart parameters measured. Expression of *bmm* in myocardial progenitor cells (*TinD*-Gal4>UAS-bmm) was similarly protective of heart function except in the progeny of NFD-fed parents that were acutely fed a HFD, which showed small decreases in diastolic diameter and heart period. (e) Cumulative incidence of three distinct cardiac defects: partial conduction blocks (non-uniform heart rate along the heart tube) non-contractile myocardial cells/regions along the heart tube, and dysfunctional ostia (malfunctioning inflow tracks during heart contractions) in the flies shown in (a–d). *bmm* expression in the fat body protected against the adverse effects of a HFD independently of parental and acute dietary exposure. (f–i) Fractional shortening (f), diastolic diameter (g), systolic diameter (h), and heart period (i) in G1 flies were significantly decreased by acute or parental exposure to a HFD. Expression of *bmm* in adult adipose tissue (*Lsp2*-Gal4>UAS-bmm) protected against the adverse effects on all heart parameters measured. Expression of *bmm* from embryonic to adulthood in adipose tissue (*ppl*-Gal4>UAS-bmm) was similarly protective except in the progeny of NFD-fed parents that were subsequently fed a HFD, which showed decreases in all parameters. (j) Cumulative incidence of the indicated cardiac defects in the flies shown in (f–i). N=18–35, ***$p < 0.001$, chi-square test for heart dysfunction and one-way ANOVA for heart parameters. Heart parameters are expressed as mean ± SEM.

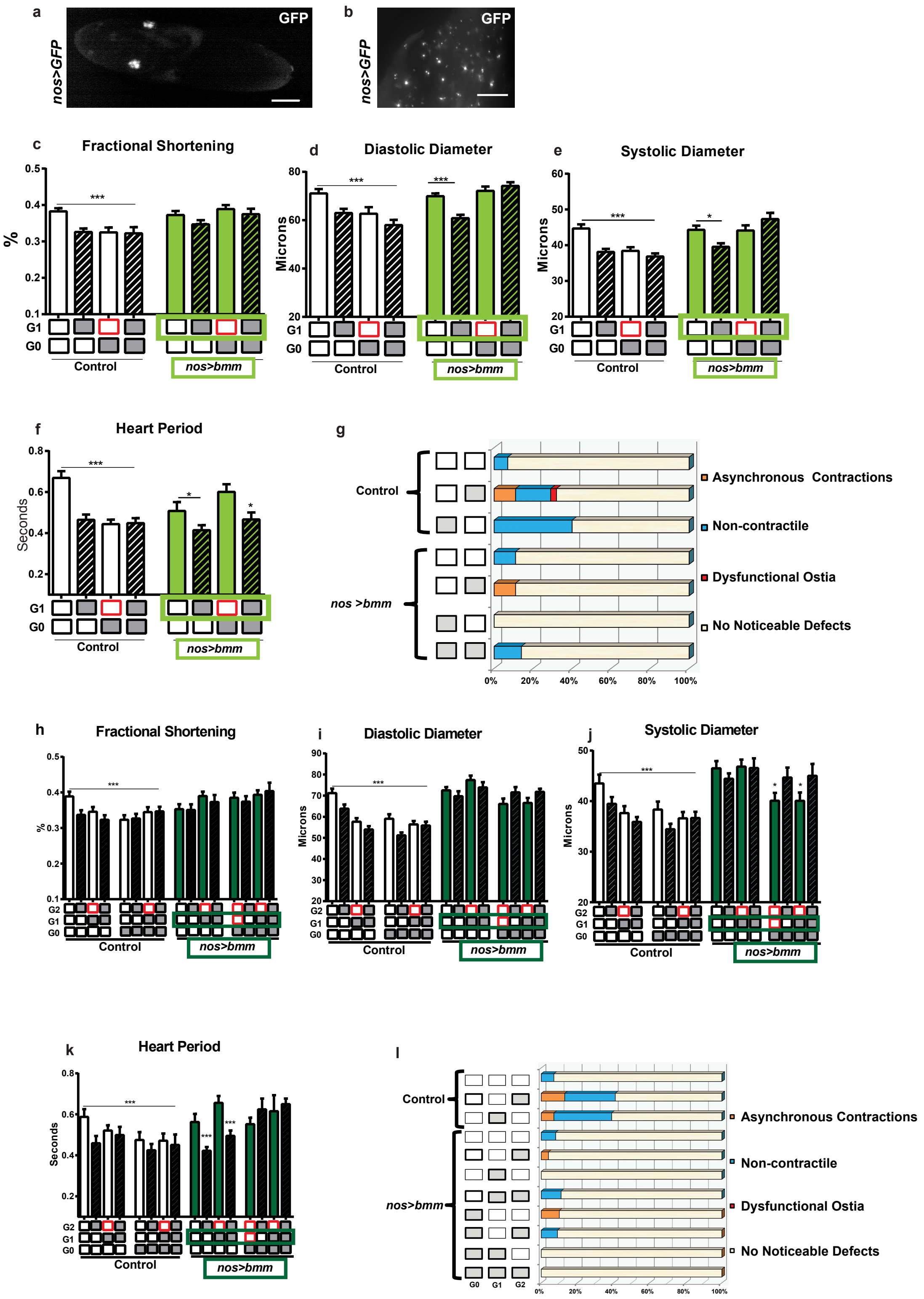

Supplementary Figure 4. Expression of *bmm* in early embryonic stage and progenitor's germ cells prevents flies from developing lipotoxic cardiomyopathy upon parental or acute exposure to a HFD. (a-b) representative immunostainings

showing GFP expression in the germ cells in the embryo (a) and nurse cells in the ovaries (b) driven by *nos-Gal4*. Scale bars are 100μm. (c-g) Fractional shortening (c), diastolic diameter (d), systolic diameter (e), and heart period (f) in G1 control flies are significantly decreased by acute or parental exposure to a HFD. Expression of the *nos*-Gal4>UAS-*bmm* transgene protected against the adverse effects of acute and parental exposure to a HFD on fractional shortening. There was a decrease in the diastolic diameter (d) and systolic diameter (e) in the *nos*-Gal4>UAS-bmm progeny of NFD-feed parents that were acutely fed a HFD. Heart period was decreased in the *nos*-Gal4>UAS-*bmm* flies after acute but not parental exposure to a HFD. (g) Cumulative incidence of heart dysfunction measurements for the flies shown in (c-f). *bmm*-expressing flies were protected against the adverse effects of a HFD independently of parental and acute dietary exposure. (h–l) Fractional shortening (h), diastolic diameter (i), systolic diameter (j), and heart period (k) are decreased in control G2 flies following acute or parental exposure to a HFD. Expression of the *nos*-Gal4>UAS-*bmm* protected flies against the effects of acute or parental exposure to a HFD on fractional shortening and diastolic diameter. A slight but significant decrease in Systolic Diameter was seen in the progeny from HFD-feed parents that were fed a NFD as adults. (k) Heart period was measured in the same flies as h-j and only flies exposed to NFD in G0 exhibited a decrease in heart period in G2 due to parental and acute HFD exposure. (l) Cumulative incidence of heart defects in the flies shown in (h–k). Flies fed a HFD in G0, G1 or G2 (acute HFD) show an increase in heart dysfunctions, which is rescued by *bmm* expression in G1. Early embryonic expression of *bmm* protected against the adverse effects of a HFD independently of parental and acute dietary exposure. Heart parameters are expressed as mean ± SEM. N=17–32, ***$p < 0.001$, chi-square test for heart dysfunction and one-way ANOVA for heart parameters.

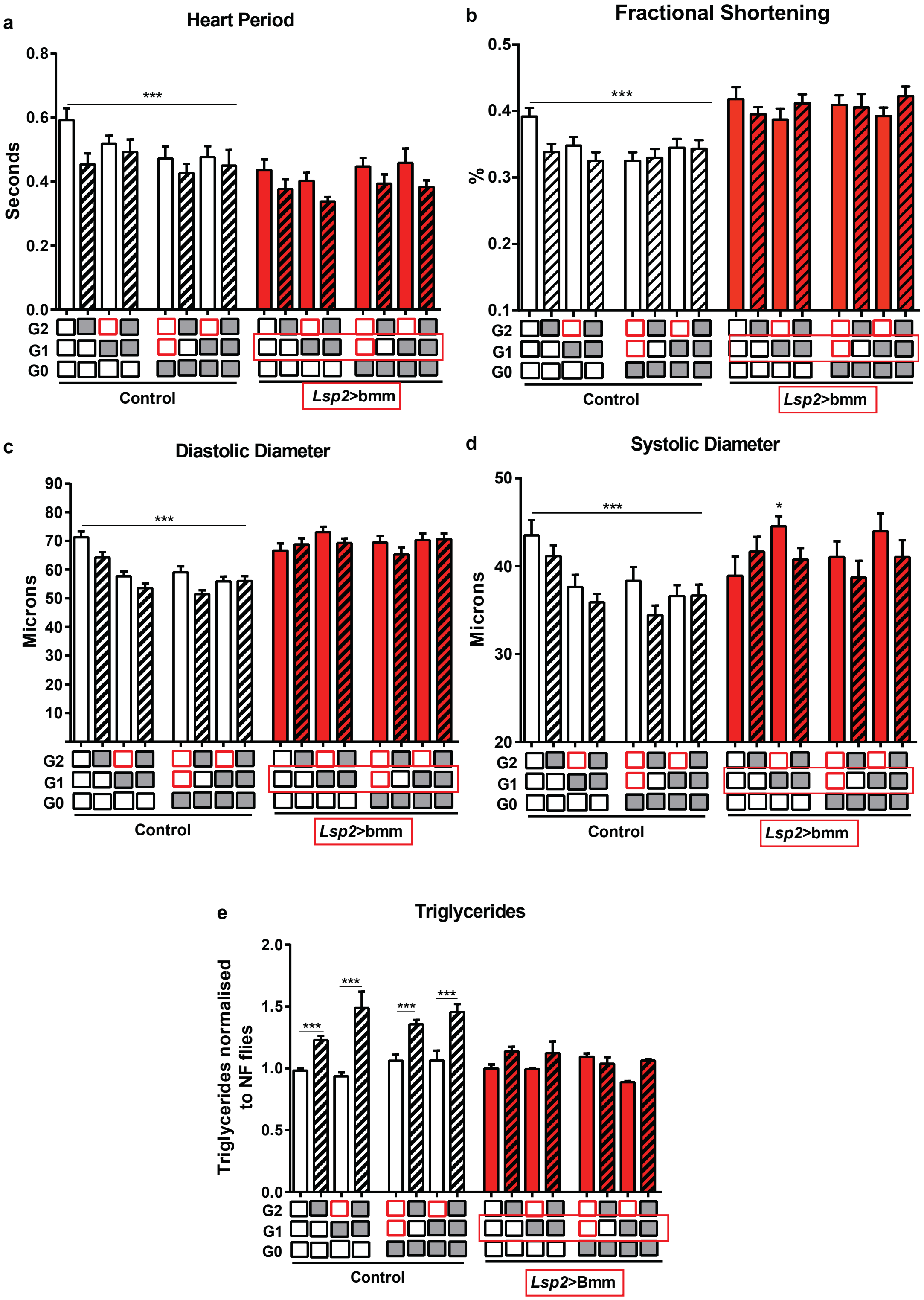

Supplementary Figure 5: Adipose-restricted expression of ATGL/*bmm* lipase protects the heart not only from parental or acute HFD in G1 (see Fig. 4c,d and S4f-j), but the next generation (G2) as well. (a-d) Heart period (a), fractional shortening (b),

diastolic diameter (c), and systolic diameter (d) are decreased in control G2 flies following acute or parental exposure to a HFD. Expression of bmm using *Lsp2*-Gal4 protected flies against the effects of acute or parental exposure to a HFD on heart period, fractional shortening and diastolic diameter. (e) TAG levels of flies exposed to parental and/or acute HFD upon expression of *bmm* in adipose fat body (*Lsp2*-Gal4>UAS-*bmm*). Adipose expression of *bmm* protected against the adverse effects of a HFD prospectively in the next generation. Note that the second-generation progeny (G2) were also protected against HFD-associated TAG accumulation when the first progeny generation (G1) expresses *bmm* in the adipose fat body. Heart parameters and TAG content are expressed as mean ± SEM. N=16–26, ***$p < 0.001$, chi-square test for heart dysfunction and one-way ANOVA for heart parameters and TAG content.

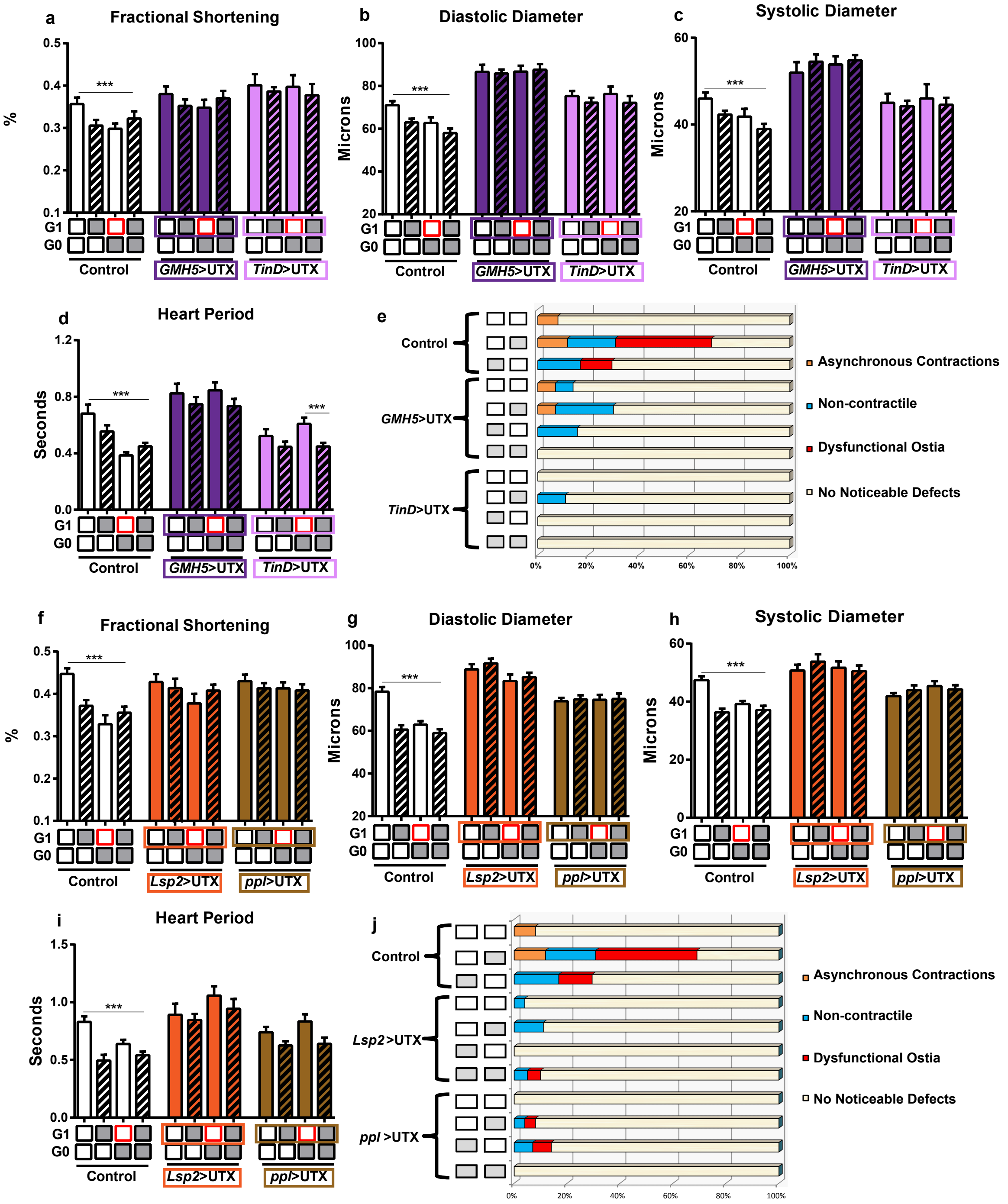

Supplementary Figure 6. Cardiac and fat body specific expression of the H3K27me3 demethylase dUTX prevents flies from acute or parental HFD effects on heart function. (a–d) Fractional shortening (a), diastolic diameter (b), systolic diameter (c), and heart period (d) in control G1 flies are significantly decreased by acute or parental exposure to a HFD. Expression of *dUTX* in adult myocardial cells (*GMH5*-Gal4>UAS-dUTX) fully protected against the adverse effects of acute or parental exposure to a HFD on heart function. *dUTX* expression in myocardial progenitor cells (*TinD*-Gal4>UAS-

dUTX) was similarly protective, except for heart period decrease in flies with acute exposure to a HFD. (e) Cumulative incidence of cardiac defects in the flies shown in (a-d). Cardiac expression of *dUTX* protected against the adverse effects of a HFD on heart function independently of parental and acute dietary exposure. (f–i) Fractional shortening (f), diastolic diameter (g), systolic diameter (h), and heart period (i) in control G1 flies are significantly decreased by acute or parental exposure to a HFD. Expression of *dUTX* in the adipose tissue using either *Lsp2*-Gal4 or *ppl*-Gal4 drivers fully protected heart function against the adverse effects of acute and parental exposure to a HFD. (j) Cumulative incidence of cardiac defects in the flies shown in (f–i). Adipose-specific expression of *dUTX* protected against the adverse effects of a HFD independently of parental and acute dietary exposure. Heart parameters are expressed as mean ± SEM. N=17–35, ***$p < 0.001$, chi-square test for heart dysfunction and one-way ANOVA for heart parameters.

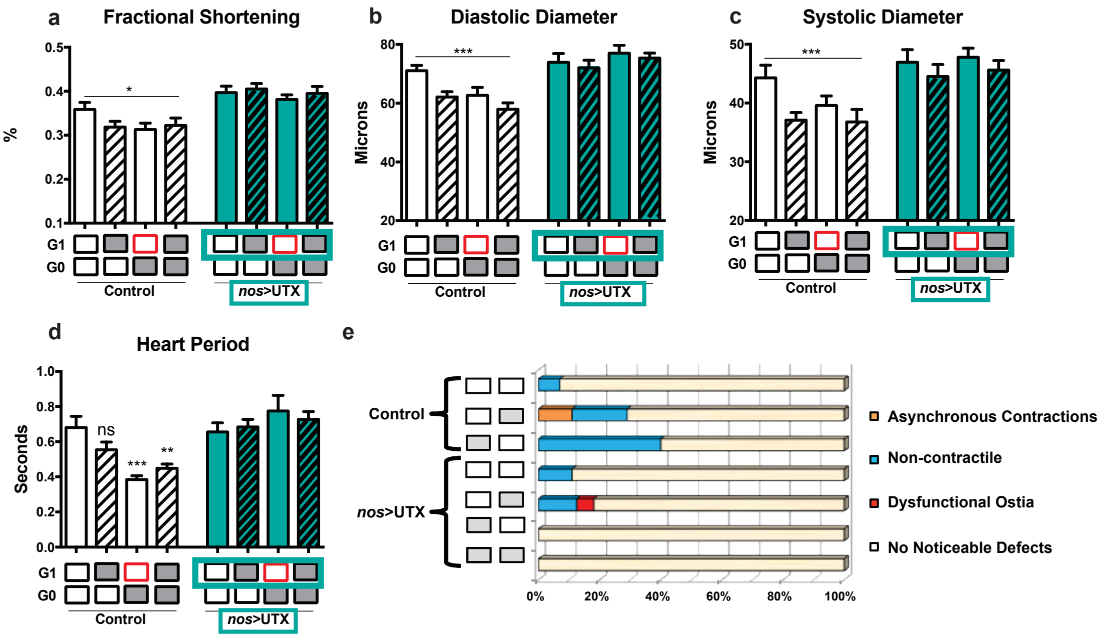

Supplementary Figure 7: expression of the H3K27me3 demethylase *dUTX* in early embryonic stage and progenitor's germ cells prevents flies from acute or parental HFD effects on heart function. (a–d) Fractional shortening (a), diastolic diameter (b), systolic diameter (c), and heart period (d) in control G1 flies are significantly decreased by acute or parental exposure to a HFD. Expression of *dUTX* using *nos*-Gal4 driver fully protected against the adverse effects of acute or parental exposure to a HFD on heart function. (e) Cumulative incidence of cardiac defects in the flies shown in (a-d). Heart parameters are expressed as mean ± SEM. N=15–20, ***$p < 0.001$, chi-square test for heart dysfunction and one-way ANOVA for heart parameters.

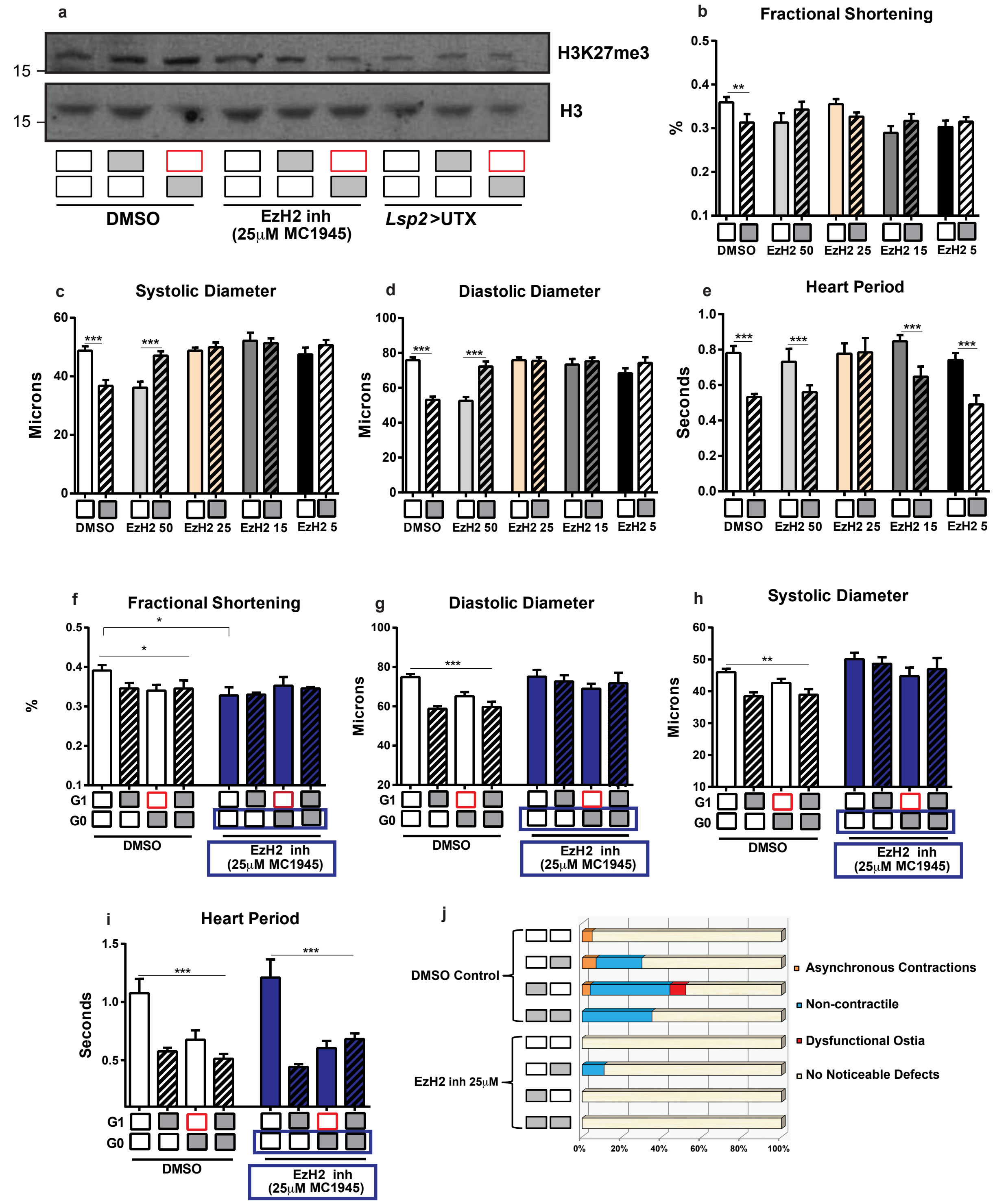

Supplementary Figure 8. Pharmacological inhibition of EzH2 reduces H3K27me3 levels and prevents HFD-induced lipotoxic cardiomyopathy. (a) Representative Western blot of H3K27m3 and total H3 from whole flies histone extract (See Fig. 6a and

7a for densitometric quantification of 2-4 independent experiments). (b-e) Dose-response curves showing the effect of the EzH2 inhibitor MC1945 (EzH2 inh) on fractional shortening (b), diastolic diameter (c), systolic diameter (d), and heart period (e). DMSO or MC1945 (100 µl of the indicated concentrations [µM]) were added to the NFD and HFD food and flies were tested for changes in heart function 5 days later. No significant changes were observed in fractional shortening at any concentration of MC1945 upon HFD feeding. Diastolic and systolic diameters were significantly increased by 50 µM MC1945 on a HFD but not by lower concentrations. A decrease in heart period was seen at all concentrations except 25 µM MC1945. A concentration of 25 µM was selected because it gave the most consistent protective phenotype against the adverse effects of acute exposure to a HFD (two-tailed unpaired t-test analysis *$p<0.05$, **$p<0.01$, ***$p<0.001$). (f–j) Populations of flies were fed for 5 days with NFD or HFD mixed with either DMSO or 25 µM MC1945 EzH2 inhibitor. Flies were then segregated, placed on NFD, and allowed to lay eggs. The embryos were grown to adulthood on NFD and then exposed to 5 days feeding with a NFD or a HFD in the absence of inhibitor. Fractional shortening (f), diastolic diameter (g), systolic diameter (h), and heart period (i) were then measured. Inhibition of EzH2 activity with 25 µM MC1945 induced a baseline decrease in fractional shortening, compared to NFD on DMSO, however there was no decrease due to HFD exposure seen in any flies from parents fed MC1945. The treatment of G0 with EzH2 inhibitor protected the next generation against the adverse effects of acute and parental HFD on diastolic and systolic diameters but not on heart period. (j) Cumulative incidence of cardiac defects in the flies shown in (f-i). When compared to the DMSO controls the EzH2 inhibitor protected the progeny against the adverse effects, on heart function, of acute or parental exposure to a HFD. Heart parameters are expressed as mean ± SEM. N=15–30, ***$p< 0.001$, chi-square test for heart dysfunction and one-way ANOVA for heart parameters.
