# Supplementary material for: Intergenerational inheritance of high fat diet-induced cardiac lipotoxicity in Drosophila
Source: Nat Commun. 2019 Jan 14;10:193. doi: 10.1038/s41467-018-08128-3 (PMC6331650; doi:10.1038/s41467-018-08128-3)
Supplement: Supplementary file 2 — Reporting Summary [file 41467_2018_8128_MOESM2_ESM.pdf]

nature research

Corresponding author(s): Rolf Bodmer

Reporting Summary

Nature Research wishes to improve the reproducibility of the work that we publish. This form provides structure for consistency and transparency in reporting. For further information on Nature Research policies, see Authors & Referees and the Editorial Policy Checklist.

Statistical parameters

When statistical analyses are reported, confirm that the following items are present in the relevant location (e.g. figure legend, table legend, main text, or Methods section).

n/a	Confirmed	
☐	☒	The exact sample size (n) for each experimental group/condition, given as a discrete number and unit of measurement
☐	☒	An indication of whether measurements were taken from distinct samples or whether the same sample was measured repeatedly
☐	☒	The statistical test(s) used AND whether they are one- or two-sided<br>*Only common tests should be described solely by name; describe more complex techniques in the Methods section.*
☒	☐	A description of all covariates tested
☐	☒	A description of any assumptions or corrections, such as tests of normality and adjustment for multiple comparisons
☐	☒	A full description of the statistics including central tendency (e.g. means) or other basic estimates (e.g. regression coefficient) AND variation (e.g. standard deviation) or associated estimates of uncertainty (e.g. confidence intervals)
☐	☒	For null hypothesis testing, the test statistic (e.g. F, t, r) with confidence intervals, effect sizes, degrees of freedom and P value noted<br>*Give P values as exact values whenever suitable.*
☒	☐	For Bayesian analysis, information on the choice of priors and Markov chain Monte Carlo settings
☒	☐	For hierarchical and complex designs, identification of the appropriate level for tests and full reporting of outcomes
☒	☐	Estimates of effect sizes (e.g. Cohen's d, Pearson's r), indicating how they were calculated
☐	☒	Clearly defined error bars<br>*State explicitly what error bars represent (e.g. SD, SE, CI)*

Our web collection on statistics for biologists may be useful.

Software and code

Policy information about availability of computer code

Data collection	SpectraMax M2e software was used to read absorbances. HCImageLive was used to image the beating hearts. Zen 2.3 pro was used to acquire images of fixed hearts and embryos.
Data analysis	SOHA software was used to analyze heart function. ImageJ was used for image processing. Statistical analysis was conducted using GraphPad. Figures were generated using Adobe Illustrator CS5.

For manuscripts utilizing custom algorithms or software that are central to the research but not yet described in published literature, software must be made available to editors/reviewers upon request. We strongly encourage code deposition in a community repository (e.g. GitHub). See the Nature Research guidelines for submitting code & software for further information.

Data

Policy information about availability of data

All manuscripts must include a data availability statement. This statement should provide the following information, where applicable:

- Accession codes, unique identifiers, or web links for publicly available datasets
- A list of figures that have associated raw data
- A description of any restrictions on data availability

The authors declare that the data supporting the findings of this study are available in the article and its Supplementary Information File. All other relevant data supporting the findings of this study are available from the corresponding author upon reasonable request.

Field-specific reporting

Please select the best fit for your research. If you are not sure, read the appropriate sections before making your selection.

☒ Life sciences ☐ Behavioural & social sciences ☐ Ecological, evolutionary & environmental sciences

For a reference copy of the document with all sections, see nature.com/authors/policies/ReportingSummary-flat.pdf

Life sciences study design

All studies must disclose on these points even when the disclosure is negative.

Sample size	For heart function parameters, a sample size of 15-20 flies was calculated to be adequate given the standard deviation, as depicted in the methods. Sample size was not predetermined using any statistical method in the case of embryo analysis.
Data exclusions	Outliers were identified using the ROUT method, Q=1% (GraphPad Prism).
Replication	All data presented here are representative of at least two independent experiments as indicated in the manuscript.
Randomization	Among same genotype, flies were randomly assigned a high fat diet or a normal food diet. Only mated female flies were used in the analysis. All collected embryos were used in this study.
Blinding	Flies were assigned a numerical code per genotype/treatment so investigators were blinded when analysing heart function.

Reporting for specific materials, systems and methods

Materials & experimental systems

n/a	Involved in the study	
☒	☐	Unique biological materials
☐	☒	Antibodies
☒	☐	Eukaryotic cell lines
☒	☐	Palaeontology
☒	☐	Animals and other organisms
☒	☐	Human research participants

Methods

n/a	Involved in the study	
☒	☐	ChIP-seq
☒	☐	Flow cytometry
☒	☐	MRI-based neuroimaging

Antibodies

Antibodies used	anti-GFP -1020 (1:1000, Aves lab); anti-alpha-spectrin (1:50, DSHB #3A9); anti-H3K27me3 (1:1000, Active Motif, #39155); anti-H3 (1:1000, Millipore, #06-755)
Validation	anti-GFP and anti-alpha-spectrin have been vastly used and validated by immunohistochemistry also in Drosophila. We have included the refences in the methods section. anti-H3K27me3 was previously used in Drosophila as depicted in the methods section and has 127 citations in different animal models as well as the anti-H3 antibody from Millipore which has 171 citations in different animal models and diverse applications including western blot.
